# The global impact of non-communicable diseases on macro-economic productivity: a systematic review

**DOI:** 10.1007/s10654-015-0026-5

**Published:** 2015-04-03

**Authors:** Layal Chaker, Abby Falla, Sven J. van der Lee, Taulant Muka, David Imo, Loes Jaspers, Veronica Colpani, Shanthi Mendis, Rajiv Chowdhury, Wichor M. Bramer, Raha Pazoki, Oscar H. Franco

**Affiliations:** Department of Epidemiology, Erasmus MC, University Medical Center Rotterdam, Office NA29-16, PO Box 2040, 3000 CA Rotterdam, The Netherlands; Department of Endocrinology, Erasmus MC, Rotterdam, The Netherlands; Department of Public Health, Erasmus MC, Rotterdam, The Netherlands; Division of Infectious Disease Control, Municipal Public Health Service (GGD) Rotterdam-Rijnmond, Rotterdam, The Netherlands; Chronic Diseases Prevention and Management, Department of Chronic Diseases and Health Promotion, World Health Organization, Geneva, Switzerland; Department of Public Health and Primary Care, University of Cambridge, Cambridge, UK; Medical Library, Erasmus MC, Rotterdam, The Netherlands

**Keywords:** Noncommunicable diseases, Productivity, Return to work absenteeism, Systematic review

## Abstract

Non-communicable diseases (NCDs) have large economic impact at multiple levels. To systematically review the literature investigating the economic impact of NCDs [including coronary heart disease (CHD), stroke, type 2 diabetes mellitus (DM), cancer (lung, colon, cervical and breast), chronic obstructive pulmonary disease (COPD) and chronic kidney disease (CKD)] on macro-economic productivity. Systematic search, up to November 6th 2014, of medical databases (Medline, Embase and Google Scholar) without language restrictions. To identify additional publications, we searched the reference lists of retrieved studies and contacted authors in the field. Randomized controlled trials, cohort, case–control, cross-sectional, ecological studies and modelling studies carried out in adults (>18 years old) were included. Two independent reviewers performed all abstract and full text selection. Disagreements were resolved through consensus or consulting a third reviewer. Two independent reviewers extracted data using a predesigned data collection form. Main outcome measure was the impact of the selected NCDs on productivity, measured in DALYs, productivity costs, and labor market participation, including unemployment, return to work and sick leave. From 4542 references, 126 studies met the inclusion criteria, many of which focused on the impact of more than one NCD on productivity. Breast cancer was the most common (n = 45), followed by stroke (n = 31), COPD (n = 24), colon cancer (n = 24), DM (n = 22), lung cancer (n = 16), CVD (n = 15), cervical cancer (n = 7) and CKD (n = 2). Four studies were from the WHO African Region, 52 from the European Region, 53 from the Region of the Americas and 16 from the Western Pacific Region, one from the Eastern Mediterranean Region and none from South East Asia. We found large regional differences in DALYs attributable to NCDs but especially for cervical and lung cancer. Productivity losses in the USA ranged from 88 million US dollars (USD) for COPD to 20.9 billion USD for colon cancer. CHD costs the Australian economy 13.2 billion USD per year. People with DM, COPD and survivors of breast and especially lung cancer are at a higher risk of reduced labor market participation. Overall NCDs generate a large impact on macro-economic productivity in most WHO regions irrespective of continent and income. The absolute global impact in terms of dollars and DALYs remains an elusive challenge due to the wide heterogeneity in the included studies as well as limited information from low- and middle-income countries.

**Electronic supplementary material:**

The online version of this article (doi:10.1007/s10654-015-0026-5) contains supplementary material, which is available to authorized users.

## Introduction

Non-communicable diseases (NCDs), such as coronary heart disease (CHD), stroke, chronic obstructive pulmonary disease (COPD), cancer, type 2 diabetes and chronic kidney disease (CKD) currently constitute the number one cause of morbidity and mortality worldwide, claiming 36 million lives each year (accounting for 63 % of all adult deaths) [1]. Infectious disease prevention and control, economic growth, improvements in medical and scientific knowledge, and health and social systems development have all contributed to increased life expectancy, improved quality of life and increased likelihood of living to age 60 years and beyond. While these are notable achievements, together with lifestyle-related shifts, these epidemiological and socio-demographic changes also mean that the burden of NCDs will grow [2].

Productivity is a measure of the efficiency of a person, business or country in converting inputs into useful outputs. The productive age span of a person is from adulthood to retirement and ranges from 18 years to around 65 years of age depending on, amongst other things, profession and country. The measurement of productivity greatly relies on the output and the economic or social system context. The focus in this report is macro-economic productivity loss in the productive age range due to NCDs. Key macro-economic measures related to the labor market include: (un-) employment, (loss in) hours worked (including full or part-time work status change), presenteeism (defined as impaired performance while at work), absenteeism, disability adjusted life years (DALYs) and productivity costs/losses. Key macro-economic outcomes are reduction in the able workforce, NCD-related health and welfare expenditure and loss of income earned by the productive workforce. While both the burden of NCDs and the socio-economic contexts vary greatly, the impact of the former on macro-economic outcomes across the global regions remains unclear.

We aimed to systematically identify and summarize the literature investigating the impact of six NCDs (CHD, stroke, COPD cancer, type 2 diabetes and CKD) on macro-economic productivity and to determine directions for future research.

## Methods

### Search strategy and inclusion criteria

**Fig. 1:**
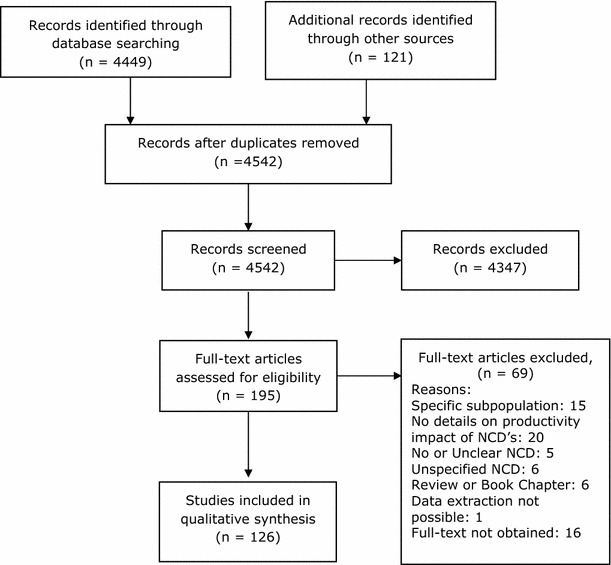
Flowchart of studies for the global impact of non-communicable diseases on macro-economic productivity

We systematically searched the electronic medical databases (Medline, Embase and Google Scholar) up to November 6th, 2014 (date of last search) to identify relevant articles evaluating the macro-economic consequences of the six selected NCDs, specifically the impact on economic productivity of working age citizens. The complete search strategy is available in “Appendix 1”. We defined the major NCDs of interest as CHD, stroke, chronic obstructive lung disease (COPD), type 2 diabetes mellitus (DM), cancer (lung, colon, breast and cervical) and chronic kidney disease (CKD). The step-wise inclusion and exclusion procedure is outlined in Fig. 1. Eligible study design included randomized controlled trials (RCTs), cohort, case–control, cross-sectional, systematic reviews, meta-analysis, ecological studies and modeling studies. We included studies that estimated the impact of at least one of the NCDs defined above on at least one of the following measures of macro-economic productivity: DALYs, economic costs related to reduced work productivity, absenteeism, presenteeism, (un) employment, (non-) return to work (RTW) after sickness absence and medical/sick leave. DALY is also considered as essentially it is an economic measure of human productive capacity for the affected individual and when taken together (e.g. all those in a company, society etc.) forms an economic measure also on the group level. Only studies involving adults (>18 years old) were included, without any restriction on language or date.

### Study selection

Two independent reviewers screened the titles and abstracts of the initially identified studies to determine if they satisfied the selection criteria. Any disagreements were resolved through discussion and consensus, or by consultation with a third reviewer. In order to ensure that all retrieved full texts (of the selected abstracts) satisfied the inclusion criteria appropriately, they were further assessed by two independent reviewers. We further screened the reference lists of all retrieved studies to retrieve relevant articles. Systematic reviews were not included in the data extraction but a supplementary scan of their reference lists was performed to identify any additional studies.

### Data extraction

A data collection form (DCF) was prepared to extract the relevant information from the included full texts, including study design, World Health Organization (WHO) region, participants, NCD-related exposure and macro-economic outcome characteristics. When evaluating economic costs, US dollars (USD) was used as outcome measure. If a study reported costs in another currency, the corresponding exchange rate to USD as reported by the study itself was used. However, if an exchange rate was not provided, we calculated USD applying the conversion rate for the indicated study time-period.

### Quality evaluation

To evaluate the quality of the included non-randomized studies, we applied the Newcastle–Ottawa Scale (NOS) [3]. The NOS scale assesses the quality of articles in three domains: selection, comparability and exposure. ‘Selection’ assesses four items and a maximum of one star can be awarded for each item. ‘Comparability’ awards a maximum of two stars to the one item within the category. Finally, ‘exposure’ includes four items for which one star can be awarded. A quality score is made for each study by summing the number of stars awarded, and thus the NOS scale can have maximum of nine stars. We used this scale to assess the quality of case–control and cohort studies. For cross-sectional and descriptive studies, we used an adapted version of NOS scale (“Appendix 2”).

### Statistical methods

We aimed to pool the results using a random effects model. If pooled, results would be expressed as pooled relative risks with 95 % confidence intervals. Pooling possibility was conditional on the level of heterogeneity between studies.

## Results

### General characteristics of the included studies

From 4542 references initially identified, a total of 126 unique studies met the inclusion criteria (Fig. 1; Table 1). All eligible studies were published between 1984 and 2014. Of the 126 studies identified, 52 were from the WHO European Region, 53 from the Region of the Americas (of which all but two were from Canada or the United States of America [USA]), 16 from the Western Pacific Region, four were from the WHO African Region and one from the Eastern Mediterranean Region. We found no studies from South East Asia. The majority of the identified studies were observational in design, analyzed prospectively as well as cross-sectional. Two studies reported cross-sectional data from an RCT and six were modeling studies. National or hospital-based disease registries were often used to select patients, which were in some cases linked to national socio-economic databases to extract corresponding employment data. The control group, if used, was often a sample from the general population and sometimes sought within the same environment of the patients (e.g. same company). Many studies focused on the impact of more than one NCD on productivity. Most studies used one measure of productivity. Of all the published studies including cancers, cervical cancer was included in seven studies, breast cancer in 45, colon cancer in 24 and lung cancer in 16. Stroke was included in a total of 31 studies, COPD in 24, DM in 22 and CHD was included in 15 studies. Relevant data on CKD was included in only two of the studies and two of the studies mention NCDs in general.

**Table 1:** General characteristics of the included studies

Source	Period of surveillance	Location	WHO region	Study design	Number in analysis	Gender	Ethnicity	Reported NCDs
Adepoju et al. [71]	2007–2012	USA	RA	Retrospective	376	Both	Hispanic, non-Hispanic black, non-Hispanic white	DM
Ahn et al. [31]	1993–2002	South Korea	WPR	Cross-sectional	1594	Female	NR	Breast cancer
Alavinia and Burdorf [69]	2004	10 EU countries	ER	Cross-sectional	11,462	Both	NR	CVD, stroke, DM
Alexopoulos and Burdorf [54]	1993–1995	The Netherlands	ER	Prospective cohort	326	Male	NR	COPD
Anesetti-Rothermel and Sambamoorthi [10]	2007	USA	RA	Cross-sectional	12,860	Both	White, Latino, African American, other	COPD, CVD, stroke, DM
Angeleri et al. [80]	NR	Italy	ER	Prospective study	180	Both	NR	Stroke
Arrossi et al. [23]	2002–2004	Argentina	RA	Cross-sectional	120	Female	NR	Cervical cancer
Bains et al. [44]	2008–2009	UK	ER	Prospective cohort	50	Female	NR	Colon cancer
Balak et al. [34]	2001–2007	The Netherlands	ER	Retrospective cohort	72	Female	NR	Breast cancer
Bastida and Pagan [81]	1994–1999	USA	RA	Population based	1021	Both	Mexican Americans	DM
Black-Schaffer and Osberg [82]	1984–1986	USA	RA	Prospective study	79	Both	NR	Stroke
Bogousslavsky and Regli [83]	NR	Switzerland	ER	Prospective study	41	Both	NR	Stroke
Boles et al. [84]	2001	USA	RA	Cross-sectional	2264	Both	NR	DM
Bouknight et al. [37]	2001–2002	USA	RA	Prospective study	416	Female	White, black	Breast Cancer
Bradley and Bednarek [85]	1999	USA	RA	Cross-sectional	184	Both	Caucasian, African-American, Hispanic, other	Breast cancer, colon cancer, lung cancer
Bradley et al. [86]	1992	USA	RA	Retrospective study	5974	Female	Caucasian, African-American, Hispanic, other	Breast cancer
Bradley et al. [87]	1992	USA	RA	Cross-sectional	5728	Female	Caucasian, African-American, Hispanic, other.	Breast cancer
Bradley et al. [88]	2001–2002	USA	RA	Prospective study	817	Female	Non-Hispanic White, Non-Hispanic African American, other	Breast cancer
Bradley et al. [89]	2001–2002	USA	RA	Prospective study	239	Female	Non-Hispanic White, Non-Hispanic African American, other	Breast cancer
Bradley and Dahman [33]	2007–2011	USA	RA	Cross-sectional	828	Both	Non-Hispanic white, non-Hispanic black, other	Breast cancer
Bradley et al. [40]	2005	USA	RA	Modelling study	NR	Both	NR	Colon cancer
Bradshaw et al. [66]	2000–2000	South Africa	AR	Modelling	NR	Both	NR	DM
Broekx et al. [90]	1997–2004	Belgium	ER	Cost–of–Illness analysis	20,439	Female	NR	Breast cancer
Burton et al. [91]	2002	USA	RA	Survey	16,651	Both	NR	DM
Carlsen et al. [45]	2001–2009	Denmark	ER	Epidemiological	4343	Both	NR	Colon cancer
Carlsen et al. [29]	2001–2011	Denmark	ER	Cross-sectional and propective	14,750	Female	NR	Breast cancer
Catalá-López et al. [13]	2008	Spain	ER	Cross-sectional	37,563,454	Both	NR	Stroke
Choi et al. [42]	2001–2003	South Korea	WPR	Prospective cohort	305	Male	NR	Colon cancer
Collins et al. [92]	2002	USA	RA	Survey	7797	Both	NR	DM
Costilla et al. [22]	2006	New Zealand	WPR	Modelling	NR	Both	Maori and non-Maori	Breast cancer, colon cancer, lung cancer, cervical cancer
Dacosta DiBonaventura et al. [53]	2009	USA	RA	Cross-sectional	20,024	Both	Non-Hispanic White, Non-Hispanic Black/African-American, Hispanic, other	COPD
Dall et al. [68]	2007–2007	USA	RA	Modelling	NR	NR	NR	DM
Darkow et al. [63]	2001–2004	USA	RA	Case–control	4045	Both	NR	COPD
De Backer et al. [93]	1994–1998	Belgium	ER	Prospective cohort	15,740	Both	NR	DM
Eaker et al. [94]	1993–2003	Sweden	ER	Cross-sectional	28,566	Female	NR	Breast Cancer
Earle et al. [46]	2003–2005	USA	RA	Prospective cohort	2422	Both	Non-Hispanic white, African American, Hispanics, Asian, mixed race	Lung cancer, colon cancer
Ekwueme et al. [26]	1970–2008	USA	RA	Retrospective cohort	53,368	Female	White and Black	Breast cancer
Etyang et al. [6]	2007–2012	Kenya	AR	Prospective surveillance	18,712	Both	NR	CVD, Stroke, DM
Fantoni et al. [38]	2004–2005	France	ER	Cross-sectional	379	Female	NR	Breast cancer
Fernandez de Larrea-Baz et al. [95]	2000	Spain	ER	Ecological	40,376,294	Both	NR	Breast cancer, colon cancer, lung cancer
Ferro and Crespo [96]	1985–1992	Portugal	ER	Prospective cohort	215	Both	NR	Stroke
Fu et al. [97]	2004–2006	USA	RA	Survey	46,617	Both	White, black, Asian, other	DM
Gabriele and Renate [18]	2001–2004	Germany	ER	Prospective cohort	70	Both	NR	Stroke
Genova-Maleras et al. [4]	2008	Spain	ER	Modelling	NR	Both	NR	CVD, stroke, COPD, lung cancer, colon cancer, breast cancer, DM
Gordon et al. [47]	2003–2004	Australia	WPR	Prospective cohort	975	Both	NR	Colon cancer
Hackett et al. [19]	2008–2010	Australia	WPR	Prospective cohort	441	Both	NR	Stroke
Halpern et al. [98]	2000	USA	RA	Economical evaluation	447	Both	NR	COPD
Hansen et al. [99]	NR	USA	RA	Cross-sectional	203	Female	White and non-white	Breast cancer
Hauglann et al. [30]	1992–1996	Norway	ER	National registry cohort	3096	Female	NR	Breast cancer
Hauglann et al. [49]	1992–1996	Norway	ER	Case–control	1480	Both	NR	Colon cancer
Helanterä et al. [65]	2007	Finland	ER	Cross-sectional	2637	Both	NR	CKD
Herquelot et al. [100]	1989–2007	France	ER	Prospective cohort	20,625	Both	NR	DM
Holden et al. [52]	2004–2006	Australia	WPR	Cross-sectional	78,430	Both	NR	CVD, COPD, DM
Hoyer et al. [101]	2007–2008	Sweden	ER	Prospective cohort	651	Female	NR	Breast cancer
Jansson et al. [59]	1999	Sweden	ER	Economic evaluation	212	Both	NR	COPD
Kabadi et al. [17]	2005–2006	Tanzania	AR	Prospective surveillance study	16	Both	NR	Stroke
Kang et al. [16]	2008	South Korea	WPR	Economic Evaluation		Both	NR	Stroke
Kappelle et al. [102]	1977–1992	USA	RA	Prospective study	296	Both	White, other	Stroke
Katzenellenbogen et al. [14]	1997–2002	Western Australia	WPR	Modelling, ecologocial	68,661	Both	Indigenous; non-indigenous	Stroke
Kessler et al. [70]	1995–1996	USA	RA	Survey	2074	Both	NR	DM
Klarenbach et al. [64]	1988–1994	USA	RA	Cross-sectional	5558	Both	White, black, other	CVD, COPD, DM, CKD
Kotila et al. [103]	1978–1980	Finland	ER	Prospective	255	Both	NR	Stroke
Kremer et al. [55]	2000–2001	Australia	ER	Cross-sectional	826	Both	NR	COPD
Kruse et al. [104]	1980–2003	Denmark	ER	Cohort	2212	Both	NR	CHD
Lauzier et al. [35]	2003	Canada	RA	Prospective cohort	962	Female	NR	Breast cancer
Lavigne et al. [67]	1999–1999	USA	RA	Cross-sectional	472	Both	NR	DM
Leigh et al. [105]	1996	USA	RA	Ecological study	2,395,650	Both	NR	COPD
Leng [106]	2004–2005	Singapore	WPR	Retrospective cohort	29	NR	NR	Stroke
Lenneman et al. [107]	2005–2009	USA	RA	Survey	577,186	Both	White, black, Hispanic, Asian, other	DM
Lindgren et al. [108]	1994	Sweden	ER	Cross-sectional	393	Both	NR	Stroke
Lokke et al. [62]	1998–2010	Denmark	ER	Case–control	262,622	Both	NR	COPD
Lokke et al. [61]	1998–2010	Denmark	ER	Case–control	1,269,162	Both	NR	COPD
Lopez–Bastida et al. [15]	2004	Canary Islands, Spain	ER	Cross-sectional	448	Both	NR	Stroke
Mahmoudlou [39]	2008	Iran	EMR	Cross-sectional	72,992,154	Both	NR	Colon cancer
Maunsell et al. [32]	1999–2000	Canada	RA	Cross-sectional	57,307	Female	NR	Breast cancer
Mayfield et al. [109]	1987	USA	RA	Survey	35,000	Both	(non)African American, (non) Hispanic	DM
McBurney et al. [110]	1999–2000	USA	RA	Cross-sectional survey	89	Both	Caucasian or minority/unknown	CVD
Molina et al. [111]	2004–2005	Spain	ER	Cross-sectional	347	Both	NR	Breast cancer, colorectal cancer, lung cancer
Molina Villaverde et al. [112]	NR	Spain	ER	Cohort	96	Female	NR	Breast Cancer
Moran et al. [5]	2000–2029	China	WPR	Ecological and modelling	1,270,000,000	Both	NR	CVD
Nair et al. [113]	2000–2007	USA	RA	Economic evaluation	853,496	Both	NR	COPD
Neau et al. [114]	1990–1994	France	ER	Retrospective	67	Both	NR	Stroke
Niemi et al. [115]	1978–1980	Finland	ER	Retrospective case-series	46	Both	NR	Stroke
Nishimura and Zaher [58]	1990–2002	Japan	WPR	Modelling study	1,848,000	Both	NR	COPD
Noeres et al. [28]	2002–2010	Germany	ER	Prospective cohort	874	Female	NR	Breast cancer
Nowak et al. [60]	2001	Germany	ER	Cross-sectional	814	Both	NR	COPD
O’Brien et al. [116]	NR	USA	RA	Cross-sectional	98	Both	Caucasian and African American	Stroke
Ohguri et al. [117]	2000–2005	Japan	WPR	Cross-sectional	43	Both	NR	Lung cancer, colon cancer
Orbon et al. [56]	1998–2000	The Netherlands	ER	Cross-sectional	2010	Both	NR	COPD
Osler et al. [12]	2001–2009	Denmark	ER	Cohort	21,926	Both	NR	CVD
Park et al. [48]	2001–2006	South Korea	WPR	Cross-sectional	2538	Both	NR	Lung cancer, colon cancer, breast cancer, cervical cancer
Park et al. [118]	2001–2006	South Korea	WPR	Prospective study	1602	Both	NR	Lung cancer, colon cancer, breast cancer, cervical cancer
Peters et al. [119]	NR	Nigeria	AR	Cross-sectional	110	Both	NR	Stroke
Peuckmann et al. [120]	1989–1999	Denmark	ER	Cross-sectional	1316	Female	NR	Breast cancer
Quinn et al. [20]	1998–2008	UK	ER	Prospective Cohort	214	Both	NR	Stroke
Robinson et al. [121]	1985–1989	UK	ER	Cross-sectional	2104	Both	Caucasian, West-Indian, Asian	DM
Roelen et al. [122]	2001–2005	The Netherlands	ER	Ecological	259	Female	NR	Breast cancer
Roelen et al. [50]	2004–2006	The Netherlands	ER	Retrospective cohort	300,024	Both	NR	Lung cancer, breast cancer
Saeki and Toyonaga [123]	2006–2007	Japan	WPR	Prospective cohort	325	Both	NR	Stroke
Sasser et al. [8]	1998–2000	USA	RA	Economic evaluation	38,012	Female	NR	Breast cancer, CVD
Satariano et al. [27]	1984–1985 1987–1988	USA	RA	Cross-sectional	1011	Female	White, black	Breast cancer
Short et al. [124]	1997–1999	USA	RA	Cross-sectional	1433	Both	White, non-white, undetermined	Breast cancer
Short et al. [11]	2002	USA	RA	Cross-sectional	6635	Both	NR	CVD, stroke, COPD, DM
Sin et al. [125]	1988–1994	USA	RA	Cross-sectional	12,436	Both	White, Black, other	COPD
Sjovall et al. [36]	2004–2005	Sweden	ER	Ecological study	14,984	Both	NR	Breast cancer, colon cancer, lung cancer
Spelten et al. [126]	NR	The Netherlands	ER	Prospective cohort	235	Female	NR	Breast cancer
Stewart et al. [127]	NR	Canada	RA	Cross-sectional	378	Female	NR	Breast cancer
Strassels et al. [128]	1987–1988	USA	RA	Cross-sectional	238	Both	African American, White, other	COPD
Syse et al. [51]	1953–2001	Norway	ER	Cross-sectional population based	1,116,300	Both	NR	Breast cancer, lung cancer, colorectal cancer
Taskila-Brandt et al. [24]	1987–1988 1992–1993	Finland	ER	Cross-sectional population based	5098	Both	NR	Cervical cancer, breast cancer, colon cancer lung cancer
Taskila et al. [129]	1997–2001	Finland	ER	Cross-sectional	394	Female	NR	Breast cancer
Teasell et al. [130]	1986–1996	Canada	RA	Retrospective cohort	563	Both	NR	Stroke
Tevaarwerk et al. [43]	2006–2008	USA and Peru	RA	Cross-sectional	530	Both	Non-Hispanic whites and whites	Breast cancer, lung cancer, colon cancer
Timperi et al. [131]	2006–2011	USA	RA	Prospective cohort	2013	Female	Whites, Blacks, Hispanic, Asian, other	Breast Cancer
Torp et al. [25]	1999–2004	Norway	ER	Prospective Registry	9646	Both	NR	Cervical cancer, breast cancer, colon cancer, lung cancer
Traebert et al. [21]	2008	Brazil	RA	Modelling, ecological	NR	Both	NR	Cervical cancer, breast cancer, colon cancer, lung cancer
van Boven et al. [57]	2009	The Netherlands	ER	Economic evaluation	45,137	Both	NR	COPD
Van der Wouden et al. [132]	1978–1980	The Netherlands	ER	Cross-sectional	313	Female	NR	Breast cancer
Vestling et al. [133]	NR	Sweden	ER	Retrospective study	120	Both	NR	Stroke
Wang et al. [134]	NR	USA	RA	Cross-sectional	199	Both	NR	CVD, COPD, diabetes
Ward et al. [135]	1993–1994	USA	RA	Cross-sectional	2529	Both	Mixed ethnicities	COPD
Wozniak et al. [136]	NR	USA	RA	Retrospective study	203	Both	Whites, blacks and other	Stroke
Yaldo et al. [41]	2006–2009	USA	RA	Case–control	330	Both	NR	Colon Cancer
Yabroff et al. [137]	2000	USA	RA	Cross-sectional	496	Both	Hispanic, non-Hispanic white, non-Hispanic black, other	Breast cancer, colon cancer
Zhao and Winget [7]	2003–2006	USA	RA	Retrospective cohort	10,487	Both	NR	CVD (CHD)
Zheng et al. [9]	2004	Australia	WPR	Economic evaluation	NR	Both	NR	CVD (CHD)

*AR* African Region, *COPD* chronic obstructive pulmonary disease, *CKD* chronic kidney disease, *CVD* cardiovascular disease, *DM* diabetes mellitus, *EMR* Eastern Mediterranean Region, *ER* European Region, *NCD* no-communicable diseases, *NR* not reported, *RA* Region of the Americas, *USA* United States of America, *WHO* World Health Organization, *WPR* Western Pacific Region

### Measures of productivity

Measures of productivity impact in the available studies included DALYs, absenteeism, presenteeism, labor market (non-) participation, RTW, change in hours worked and medical/sickness leave. Most studies focused on the direct impact on the patient but a minority also examined the impact on caregivers/spouses. Outcomes were quantified using risks, proportions, odds, dollars, years and days. In some studies, time-to-event data was analyzed using Cox proportional-hazards regression. Adjusting for education, age and employment status was most frequently applied, although the measurement of education and employment was not consistently defined, measured or validated. A small minority of studies reported differences in impact according to ethnicity. Pooling of outcomes was not possible due to substantial heterogeneity across and within NCD groups (*I*^2^ > 70 %).

### Impact of cardiovascular disease on productivity

Of all DALYs on a population level in Spain (Table 2a), 4.2 % were attributable to CHD [4] with an estimated age-standardized rate of 4.7 per 1000 persons per year. In China, DALYs attributable to CHD were estimated to be 8,042,000 for the year 2000 and predicted to more than double in 2030, rising up to 16,356,000 [5]. In the same study, the estimated DALY in 2000 was 16.1 per 1000 persons and predicted to be 20.4 in 2030 (estimate not accounted for age). A study from Kenya estimated the DALY to be 68 per 100,000 person-years of observation [6]. CHD-related productivity loss in the USA was estimated to be 8539 USD per person per year (PP/PY), at 10175 USD PP/PY [7] for absenteeism and 2698 USD PP/PY for indirect work-related loss [8]. Total absenteeism-related costs in Australia were estimated at 5.69 billion USD, mortality-related costs at 23 million USD and costs related to lower employment at 7.5 billion USD [9]. An estimated 4.7 working days PP/PY were lost in the USA owing to CHD [10]. Also in the USA, the odds of experiencing limited amount of paid work due to illness were significantly higher for those with CHD compared to the control group, with an odds ratio (OR) of 2.91 for women (95 % CI 2.34–3.61) and 2.34 for men (95 % CI 1.84–2.98) [11]. In Denmark workforce participation increased with increasing time from 37 % after 30 days to 65 % after 5 years of diagnosis [12]. In a study conducted in 10 European Union (EU) countries, no difference was found for the risk of non-participation in the labor force between those with and without self-reported CHD with an OR of 0.96 (95 % CI 0.66–1.40).

**Table 2:** Results of the included studies investigating the impact of CVD on productivity

Study	Type of outcome	Outcome specified as	Assessment type	Point estimate	SD for mean	95 % CI	Quality score
*a*							
Alavinia and Burdorf [69]	Unemployment	Non-participation in the labor force	OR		NR	0.66–1.40	4
Anesetti-Rothermel and Sambamoorthi [10]	Sick leave	Work days in last year lost due to illness	Mean	4.700	7.89 (SE)	NR	6
Etyang et al. [6]	DALYs	Rate per 100,000 person year of observation	Rate	68	NR	NR	5
Genova-Maleras et al. [4]	DALYs	Rate per 1000 age standardised	Rate	4.7	NR	NR	NA
		Percentage of all causes of mortality	Percent	4.2	NR	NR	
Holden et al. [52]	Productivity Loss	Absenteeism (no. days or part days missed from work in last 4 weeks)	IRR	1.17	NR	1.03–1.32	3
		Presenteeism (self-rated score of overall performance over last 4 weeks)	IRR	1.65	NR	1.22–2.21	
Klarenbach et al. [64]	Unemployment	Non-participation in labor force	OR	1.27	NR	0.45–3.53	6
Kruse et al. [104]	Labor market participation	Labor market withdrawal a year after the disease debut (controls 7 %)	Percent	21	NR	NR	6
		Risk of labor market withdrawal	HR	1.32	NR	1.11–1.57	
McBurney et al. [110]	Return to work	Return to work at a mean of 7.5 months	Percent	76.4	NR	NR	4
	Presenteeism	Perceived work performance	Mean	3.6	0.52	NR	
Moran et al. [5]	DALYs	Observed period 2000	Count	80,420,00	NR	NR	NA
		Observed period 2000	Rate	16.1	NR	NR	
		Predicted 2010	Count	107,300,00	NR	NR	
		Predicted 2010	Rate	16.5	NR	NR	
		Predicted 2020	Count	134,220,00	NR	NR	
		Predicted 2020	Rate	18.2	NR	NR	
		Predicted 2030	Count	16356000	NR	NR	
		Predicted 2030	Rate	20.4	NR	NR	
Osler et al. [12]	Labor market participation	Workforce participation 30 days after diagnosis (among patients who were part of the workforce at time of diagnosis)	Percent	37.2	NR	NR	5
		Workforce participation 1 year after diagnosis (among patients who were part of the workforce at time of diagnosis)	Percent	40.1	NR	NR	
		Workforce participation 2 years after diagnosis (among patients who were part of the workforce at time of diagnosis)	Percent	45.0	NR	NR	
		Workforce participation 5 years after diagnosis (among patients who were part of the workforce at time of diagnosis)	Percent	65.2	NR	NR	
Sasser et al. [8]	Productivity loss costs	Attributable annual indirect work-loss costs per patient	USD	2698	NR	NR	8
Short et al. [124]	Unemployment	Limited amount of paid work possible due to illness female	OR	2.91	NR	2.34–3.61	5
		Limited amount of paid work possible due to illness male	OR	2.34		1.84–2.98	
Wang et al. [134]	Absenteeism	Annual excess in days	Mean	8.8	7.0 (SE)	NR	4
	Presenteeism	Annual excess in days	Mean	8.9	11.8 (SE)	NR	
	Absenteeism and presenteeism combined	Annual excess in days	Mean	16.3	12.7 (SE)	NR	
Zhao and Winget [7]	Productivity loss costs	Short term 1 year productivity costs/per person	USD	8539	NR	NR	6
		Absenteeism 1 year productivity costs/per person	USD	10175	NR	NR	
Zheng et al. [9]	Productivity loss costs	Absenteeism related total	USD	568,500,000	NR	NR	NA
		Mortality related	USD	235,650,00	NR	NR	
		Due to lower employment	USD	750,000,000	NR	NR	
*b*							
Alavinia and Burdorf [69]	Unemployment	Non participation in the labour force	OR	1.110	NR	0.530–2.320	4
Anesetti-Rothermel and Sambamoorthi [10]	Sick leave	Work days in last year lost due to illness	Mean	17.960	5.83 (SE)	–	6
Angeleri et al. [80]	Return to work	Return to work 12–196 months (mean 37.5) in hemiplegic patients	Percent	20.64	NR	NR	6
Black-Schaffer and Osberg [82]	Return to work	Return to work at 6–25 months post-rehabilitation	Percent	49	NR	NR	3
		Time return to work in months from rehabilitation	Mean	3.1	2.12	NR	
		Return to prior job at 6–25 months post-rehabilitation	Percent	43	NR	NR	
Bogousslavsky and Regli [83]	Return to work	Return to work 6–96 months (mean 46)	Count	19	NR	NR	3
Catalá-López et al. [13]	DALYs	Total	Count	418,052	NR	NR	4
		Male	Count	220,005	NR	NR	
		Female	Count	198,046	NR	NR	
Etyang et al. [6]	DALYs	Rate per 100,000 person year of observation	Rate	166	NR	NR	5
Ferro and Crespo [96]	Unemployment	Inactive at end of follow-up (mean 33.4 months, range 1–228 months)	Percent	27	NR	NR	4
Gabriele and Renate [18]	Return to Work	Return to work after 1 year of those employed	Percent	26.7	NR	NR	4
Genova-Maleras et al. [4]	DALYs	Rate per 1000 age standardised	Rate	3.8	NR	NR	NA
		Percentage of all causes of mortality	Percent	3.5	NR	NR	
Hackett et al. [19]	Return to work	Return to work 1 year after event	Percent	75	NR	NR	2
Kabadi et al. [17]	Return to work	Average months off work in 6 month follow up period	Mean	6	NR	NR	4
	Costs	Mean productivity losses due to stroke	USD	213	NR	NR	
Kang et al. [16]	Productivity loss costs	Male, total modelled costs per severe stroke per year	USD	537,724	NR	NR	NA
		Female, total modelled costs per severe stroke per year	USD	171,157	NR	NR	
Kappelle et al. [102]	Unemployment	Unemployment at 0.02–16 years after event (mean 6 years)	Percent	58	NR	NR	5
Katzenellenbogen et al. [14]	DALYs	Male	Count	26,315	NR	NR	NA
		Female	Count	30,918	NR	NR	
		Male, rate per 10,000 people, age standardized—indigenous	Rate	2027	NR	1909–2145	
		Female, rate per 10,000 people, age standardized—indigenous	Rate	1598	NR	1499–1697	
		Male, rate per 10,000 people, age standardized—non-indigenous	Rate	640	NR	633–648	
		Female, Rate per 10,000 people, age standardized—non-indigenous	Rate	573	NR	567–580	
Klarenbach et al. [64]	Unemployment	Non-participation in labour force	OR	2.21	NR	(0.7–7)	6
Kotila et al. [103]	Return to work	Return to work after 12 months	Percent	59	NR	NR	4
Leng [106]	Return to work	Return to work in 1 year	Percent	55.0	NR	NR	NA
Lindgren et al. [108]	Productivity loss costs	Indirect costs during one ear	USD	17,844	NR	12,275–23,864	4
Lopez-Bastida et al. [15]	Productivity loss costs	Indirect per person, 1 year after stroke	USD	2696	6462	NR	5
		Indirect per person, 2 year after stroke	USD	1393	4754	NR	
		Indirect per person, 3 year after stroke	USD	1362	4931	NR	
		Caregivers cost per person per year, 1 year after stroke	USD	14,732	14,616	NR	
		Caregivers cost per person per year, 2 year after stroke	USD	15,621	14,693	NR	
		Caregivers cost per person per year, 3 year after stroke	USD	13,759	15,470	NR	
Neau et al. [114]	Return to work	Return to work in same position as prior to stroke	Percent	54	NR	NR	3
		Return to work after 0–40 month (mean 7.8)	Percent	73	NR	NR	6
Niemi et al. [115]	Return to work	Return to work after 4 years	Percent	54	NR	NR	
O’Brien et al. [116]	Return to work	Return after 6–18 months	Percent	56.0	NR	NR	1
Peters et al. [119]	Return to work	Return to work after 3–104 months (mean 19.5)	Percent	55	NR	NR	3
Quinn et al. [20]	Return to Work	unemployment at 1 year follow up	Percent	47	NR	NR	3
Roelen et al. [122]	Return to Work	Return to work after 3–104 months (mean 19.5)	Percent	55.0	NR	NR	6
Saeki and Toyonaga [123]	Return to Work	Return to work at 18 months	Percent	55.0	NR	NR	6
Short et al. [124]	Unemployment	Limited amount of paid work possible due to illness female	OR	2.26	NR	1.56–2.26	5
		Limited amount of paid work possible due to illness male	OR	3.86	NR	2.55–3.60	
Teasell et al. [130]	Return to work	Return to work at 3 months	Percent	20	NR	NR	3
		Return to work full-time at 3 months	Percent	6	NR	NR	
Vestling et al. [133]	Return to work	Return to work mean of 2.7 years	Percent	41	NR	NR	3
		Time to return to work in months	Mean	11.9	9	NR	
		Return to work with reduced work hours	Percent	21	NR	NR	
Wozniak et al. [136]	Return to work	Return to work after 1 year	Percent	53	NR	NR	6
		Return to work after 2 year	Percent	44	NR	NR	
*c*							
Arrossi et al. [23]	Return to work	Reduced in hours worked (patients)	Percent	45	NR	NR	4
		Change of work (pat.)	Percent	5	NR	NR	
		Starting paid work (pat.)	Percent	14	NR	NR	
		Increased in hours worked (pat.)	Percent	11	NR	NR	
		Odds of work interruption (pat.)	OR	4	NR	NR	
		Odds of reduction in hours worked (pat.)	OR	1	NR	NR	
		Odds of starting paid work (pat.)	OR	2	NR	NR	
		Odds of increase in hours worked (pat.)	OR	1	NR	NR	
		Work interruption (caregivers)	Percent	3	NR	NR	
		Reduction in hours worked (caregivers)	Percent	61	NR	NR	
		Change of work (caregivers)	Percent	2	NR	NR	
		Starting paid work (caregivers)	Percent	5	NR	NR	
		Increased in hours worked (caregivers)	Percent	24	NR	NR	
		Work interruption (patients)	Percent	28	NR	NR	
Costilla et al. [22]	DALYs	Female	Count	1016	NR	NR	NA
		Percentage of all cancers, female	Percent	1.6	NR	NR	
		Rate per 10,000 people (age standardized)	Rate	84	NR	NR	
Park et al. [48]	Labour market participation	Time until job loss between patients and controls Cox PH	HR	1.32	NR	0.95–1.82	7
Park et al. [118]	Labour market participation	Time until job loss between patients and controls Cox PH	HR	1.68	NR	1.40–2.01	5
		Time until re-employment between patients and controls Cox PH	HR	0.67	NR	0.46–0.97	
Taskila-Brandt et al. [24]	Labor market participation	Employment status cancer survivors 2–3 years post-diagnosis compared to general population (58 vs. 75 %)	RR	0.77	NR	0.67–0.90	6
Traebert et al. [21]	Labor market participation	Employment in 5 years from diagnosis	OR	0.92	NR	0.63–1.34	9
Traebert et al. [21]	DALY	Rate per 10,000 people (age standardized)	Rate	118.7	NR	NR	NA
		Percentage of all cancers (in females)	Percent	13.4	NR	NR	
		Total	Count	2516.1	NR	NR	
*d*							
Ahn et al. [31]	Labour market drop-out	Not working current for cancer survivors versus the general population (adjusted)	OR	1.680	1.350	2.100	3
		OR of not working for cancer survivors of currently not working compared with their employment status at the time of diagnosis	OR	1.630	1.510	1.760	
	Unemployment	Adjusted OR for not working at the time of diagnosis versus the general population	OR	1.210	0.960	1.530	
Balak et al. [34]	Sick leave	Months to fully return to work	Mean	11.4	NR	NR	3
		Months to return to partial work	Mean	9.5	NR	NR	
Bouknight et al. [37]	Return to work	Return to work in 12 months after diagnosis	Percent	82	NR	NR	5
		Return to work in 18 months after diagnosis	Percent	83	NR	NR	
Bradley and Bednarek [85]	Unemployment	Unemployed 5–7 years after diagnosis for cancer survivors	Percent	54.8	NR	NR	5
		Unemployed 5–7 years after diagnosis for cancer survivors	Percent	45.4	NR	NR	
Bradley et al. [86]	Labor market participation	Probability of working of breast cancer patients compared to controls at mean of 7 years	Percent	−7	4	NR	8
Bradley et al. [87]	Labor market participation	Probability of working of breast cancer patients compared to controls at mean of 7.15 years	Percent	−10	4	NR	5
Bradley et al. [89]	Employment	Probability of being employed for patients compared to controls at 6 months	Percent	−25	NR	NR	7
		Reduced weekly hours of work for patients compared to controls after 6 months	Percent	−18	NR	NR	
Bradley et al. [40]	Absenteeism	Days absent from work evaluated at 6 months after diagnosis	Mean	44.5	55.2	NR	7
Bradley and Dahman [33]	Labor market participation	Probability of stopping work at 2 months post diagnosis (husbands of female patients)	OR	2.642	NR	0.848–8.225	5
	Labor market participation	Probability of stopping work at 9 months post diagnosis (husbands of female patients)	OR	0.843	NR	0.342–2.198	
	Productivity	Odds of decrease in weekly hours at 2 months post diagnosis (husbands of female patients)	OR	1.449		0.957–2.192	
	Productivity	Odds of decrease in weekly hours at 9 months post diagnosis (husbands of female patients)	OR	1.057		0.69–1.62	
	Productivity	Change in weekly hours at 2 months post diagnosis (husbands of female patients) (hours)	Count	−0.007	(0.885) SE	NR	
	Productivity	Change in weekly hours at 9 months post diagnosis (husbands of female patients) (hours)	Count	1.814	(1.261) SE	NR	
Broekx et al. [90]	Productivity	Indirect costs work per patient per year (attributable)	USD	5248	NR	NR	3
		Indirect costs housekeeping per patient per year (attributable)	USD	2034	NR	NR	
		Indirect costs mortality per patient per year (attributable)	USD	14,203	NR	NR	
		Sick leave days per year	USD	47.2	NR	NR	
		Total indirect costs per patient per year (attributable)	USD	21,485	NR	NR	
Carlsen et al. [45]	Unemployment	% of working women 2 years after treatment	Percent	72	NR	NR	5
Costilla et al. [22]	DALYs	DALYs % of all cancers	Percent	27.2	NR	NR	NA
		Rate per 10,000 people (age standardized)	Rate	1065	NR	NR	
		DALYs	Count	17,840	NR	NR	
Eaker et al. [94]	Sick leave	Percentage difference of sickness absence comparing patients 5 years after diagnosis with women without breast cancer	Percent	10.100	NR	NR	7
		Percentage difference of sickness absence comparing patients 3 years after diagnosis with women without breast cancer	Percent	11.100	NR	NR	
Ekwueme et al. [26]	Productivity loss	Mortality-related total lifetime productivity loss (whites)	USD	3,920,400,000	NR	NR	4
		Mortality-related total lifetime productivity loss (blacks)	USD	1323200000	NR	NR	
		Mortality-related total lifetime productivity loss/per death (all)	USD	1,100,000	NR	NR	
		Mortality-related total lifetime productivity loss/per death (whites)	USD	1,090,000	NR	NR	
		Mortality-related total lifetime productivity loss/per death (blacks)	USD	1,110,000	NR	NR	
		Mortality-related total lifetime productivity loss (all)	USD	5,488,600,000	NR	NR	
Fantoni et al. [38]	Return to work	Return to work 12 months after starting treatment	Percent	54.3	NR	NR	5
		Return to work after 3 years after starting treatment	Percent	82.1	NR	NR	
	Sick leave	Duration of sick leave 36 months after starting treatment in months	Mean	1.8	NR	9.2–12.1	
Fernandez de Larrea-Baz N et al. [95]	DALYs	Rate per 10,000 people, age standardized, male	Rate	2	NR	NR	4
		Rate per 10,000 people, age standardized, total	Count	77,382	NR	NR	
		Rate per 10,000 people, age standardized, female	Rate	374	NR	NR	
Genova-Maleras et al. [4]	DALYs	Rate per 1,000 people, age standardized	Rate	1.6	NR	NR	NA
		Percentage of all causes of mortality	Percent	1.4	NR	NR	
Hansen et al. [99]	Presenteeism	Average score difference on work limitation scale between cases and non-cancer controls	Mean	2.9	NR	NR	5
Hauglann et al. [30]	Unemployment	Unemployment at 9 years in females	Percent	18	NR	NR	9
Hoyer et al. [101]	Unemployment	Unemployment at follow up	Percent	26	NR	NR	4
Lauzier et al. [35]	Sick leave	Percent taking sick leave for 1 week or more	Percent	90.7	NR	NR	6
		Weeks of absence due to breast cancer	Count	32.3	NR	NR	
Maunsell et al. [32]	Unemployment	Unemployment among disease free survivors	Risk ratio	1.35	NR	1.08–1.7	7
	Unemployment	Unemployment among survivors with new breast cancer event	Risk ratio	2.24	NR	1.57–3.18	
	Unemployment	Unemployment among all survivors (3 years after diagnosis)	Risk ratios	1.46	NR	1.18–1.81	
	Productivity loss	Survivors reporting part-time working compared to controls (3 years after diagnosis)	Percent	4	NR	NR	
	Productivity loss	Change in working hours among survivors–change over time compared to controls (3 years after diagnosis)	Mean	−2.6	NR	NR	
Molina et al. [111]	Return to work	Return to work at mean time since diagnosis(32.5 months)	Percent	56	NR	NR	5
Molina Villaverde et al. [112]	Return to work	Return to work by end of treatment	Percent	56	NR	NR	NA
Noeres et al. [28]	Unemployment	6 years after diagnosis	Percent	43.2	NR	NR	5
		1 year after diagnosis	Percent	49.8	NR	NR	
Park et al. [48]	Labour market participation	Time until job loss (months)	Mean	36	NR		7
		Time until 25 % of patients were re-employment (months)	Mean	30	NR		
Park et al. [118]	Labour market participation	Cox proportional analysis comparing time until job loss between patients and controls	HR	1.83	NR	1.60–2.10	5
		Cox proportional analysis comparing time until re-employment between patients and controls	HR	0.61	NR	0.46–0.82	
Peuckmann et al. [120]	Labor market participation	Age-standardized prevalence of employment at 5–15 years post primary surgery	Percent	49	NR	NR	4
		Age standardized risk ratio (SRR) of employment at 5–15 years post primary surgery	SRR	1.02	NR	0.95–1.10	
		Age-standardized prevalence of sick leave at 5–15 years post primary surgery	Percent	12	NR	NR	
		Age standardized risk ratio (SRR) of sick leave at 5–15 years post primary surgery	SRR	1.28	NR	0.88–1.85	
Roelen et al. [50]	Return to work	Time to return to full-time work (days)	Count	349.0	NR	329–369	6
		Time to return to part-time work (days)	Count	271.0	NR	246–296	
Roelen et al. [112]	Return to work	Return to work at 2 years	Percent	89.4	NR	NR	4
	Sick leave	Days of absence due to breast cancer	Count	349	NR	NR	
Sasser et al. [8]	Productivity loss costs	Attributable annual indirect work-loss costs per female patient	USD	5944.0	NR	NR	8
Satariano et al. [27]	Return to work	3 months after diagnosis (white women)	Percent	74.2	NR	NR	3
	Return to work	3 months after diagnosis (black women)	Percent	59.6	NR	NR	
	Sick leave	3 months after diagnosis (white women)	Percent	25.8	NR	NR	
	Sick leave	3 months after diagnosis (black women)	Percent	40.4	NR	NR	
Short et al. [124]	Unemployment	The chances of quitting work/unemployment 1–5 years after diagnosis	OR	0.44	NR	0.20–0.95	5
Sjovall et al. [36]	Sick leave	Days sick leave taken before return to work	Count	90	NR	NR	5
Spelten et al. [126]	Return to work	Time to return to work after diagnosis analyzed using Cox PH	HR	0.45	NR	0.24–0.86	4
Stewart et al. [127]	Unemployment	Unemployment assessed at least at 2 years after diagnosis, mean of 9 years	Percent	41	NR	NR	3
Syse et al. [51]	Labor market participation	Employment probability in the year 2001 of cancer survivors compared to general population	OR	0.74	NR	0.65–0.84	6
Taskila-Brandt et al. [24]	Labor market participation	Employment status of cancer survivors 2–3 years post-diagnosis compared to general population (61 vs. 65 %)	RR	0.95	NR	0.92–0.98	6
Taskila et al. [129]	Work ability	Current work ability assessed between 0 and 10 by questionnaire (reference group 8.37)	Mean	8.23	NR	NR	8
Tevaarwerk et al. [43]	Unemployment	Unemployment	Percent	19.4	NR	NR	6
Timperi et al. [131]	Unemployment	6 months post diagnosis	Percent	52.0	NR	NR	4
Torp et al. [25]	Labor market participation	Employment 5 years from diagnosis	OR	0.74	NR	0.63–0.87	9
Traebert et al. [21]	DALYs	Percentage of all cancers, female	Percent	21.9	NR	NR	NA
		Rate per 10,000 people, age standardized, male	Rate	3.2	NR	NR	
		Percentage of all cancers, male	Percent	0.3	NR	NR	
		Total	Count	6032.3	NR	NR	
		Rate per 10,000 people, age standardized, female	Rate	195	NR	NR	
Van der Wouden et al. [132]	Labor market participation	Changes in employment status at least 5 years cancer free	Percent	−7	NR	NR	3
		Maintained employment status after diagnosis	Percent	16	NR	NR	
Yabroff et al. [137]	Labor market participation	Job in past 12 months, compared to control group (45.9 % with a *p* value <0.001 for difference)	Percent	36.9	NR	31.0–42.8	6
	Sick leave	Days lost from wok due to health problems in past 12 months compared to control group (5.7 % with a *p* value <0.001 for difference)	Mean	21.0	NR	28.4–58.3	
	Presenteeism	Limited in work due to health issues compared to control group (17.6 % with a *p* value of <0.001 for difference)	Percent	22.5	NR	17.4–27.6	
*e*							
Bains et al. [44]	Unemployment	6 months after surgery	Percent	61	NR	NR	2
Bradley et al. [40]	Productivity loss	Annual productivity losses total 2020 modelled (millions)	USD	21,780	NR	NR	NA
		Annual productivity losses total 2005 (millions)	USD	20,920	NR	NR	
Bradley and Bednarek [85]	Unemployment	Unemployed 5–7 years after diagnosis cancer survivors	Percent	54.8	NR	NR	5
		Unemployed 5–7 years after diagnosis spouse of cancer survivors	Percent	53	NR	NR	
Carlsen et al. [29]	Return to Work	Return to work after 1 year after diagnosis	Percent	69	NR	NR	8
Choi et al. [42]	Unemployment	Lost job at 24 months in males	Percent	46	NR	NR	7
Costilla et al. [22]	DALYs	Female	Count	8431	NR	NR	NA
		% of all cancers (Female)	Percent	12.9	NR	NR	
		Rate per 10,000 people (age standardised, Female)	Rate	333	NR	NR	
		Male	Count	8316	NR	NR	
		% of all cancers (Male)	Percent	13.5	NR	NR	
		Rate per 10,000 people (age standardised, Male)	Rate	414	NR	NR	
Earle et al. [46]	Unemployment	Unemployment at 15 months	Percent	65	NR	NR	4
Fernandez de Larrea-Baz N et al. [95]	DALYs	Rate per 10,000 people, age standardized, female	Rate	212	NR	NR	4
		Rate per 10,000 people, age standardized, male	Rate	284	NR	NR	
		Rate per 10,000 people, age standardized, total	Count	99,833	NR	NR	
Genova-Maleras et al. [4]	DALYs	Rate per 1000 people, age standardized	Rate	2.3	NR	NR	NA
		Percentage of all causes of mortality	Percent	2.1	NR	NR	
Gordon et al. [47]	Return to work	Working 1 year after diagnosis (%)	Percent	65	NR	NR	5
Hauglann et al. [49]	Return to work	% of employed that were on sick-leave at some point after 1 year of diagnosis	Percent	85			9
		Sickness absence for CRC localized, the OR is for 3 years after diagnosis	Odds Ratio	2.61	1.36	4.95	
		Sickness absence for CRC regional, the OR is for 3 years after diagnosis	Odds Ratio	1.09	0.56	2.11	
		Sickness absence for CRC distant, the OR is for 3 years after diagnosis	Odds Ratio	2.30	0.57	0.927	
Mahmoudlou [39]	DALYs	Total burden of colorectal cancer according to DALY in Iran in 2008	Count	52,534	NR	NR	8
		DALYs for men in 2008	Count	29,928	NR	NR	
		DALYs for women in 2008	Count	22,606	NR	NR	
Molina et al. [111]	Return to work	Return to work at mean time since diagnosis(32.5 months)	Percent	55	NR	NR	5
Ohguri et al. [117]	Sick leave	Attendance rate after return to work of employees with disease compared to controls (*p* value 0.67)	Percent	86	NR	NR	4
Park et al. [48]	Return to work	Time until re-employment (patients after job loss) Cox PH analysis	HR	0.96	NR	0.7–1.32	7
	Unemployment	Cox PH analysis time until job loss	HR	1.04	NR	0.91–1.2	
Park et al. [118]	Labour market participation	Cox PH analysis comparing time until job loss between patients and controls	HR	1.69	NR	1.50–1.90	5
		Cox PH analysis comparing time until re-employment between patients and controls	HR	0.57	NR	0.43–0.75	
Sjovall et al. [36]	Sick leave	Days sick leave	Count	115	NR	NR	5
Syse et al. [51]	Employment	Employment probability in year 2001 of cancer survivors compared to general population–men	OR	0.67	NR	0.58–0.78	6
		Employment probability in year 2001 of cancer survivors compared to general population–women	OR	0.74	NR	0.65–0.84	
Taskila-Brandt et al. [24]	Labor market participation	Employment status of cancer survivors 2–3 years post-diagnosis compared to general population (53 vs. 59 %)	RR	0.90	NR	0.81–0.99	6
Tevaarwerk et al. [43]	Unemployment	Unemployment	Percent	24.1	NR	NR	6
Torp et al. [25]	Labour market participation	Employment in 5 years from diagnosis (females)	OR	0.84	NR	0.53–1.35	9
		Employment in 5 years from diagnosis (male)	OR	0.7	NR	0.43–1.15	
Traebert et al. [21]	DALYs	Rate per 10,000 people, age standardized, female	Rate	82.6	NR	NR	NA
		Percentage of all cancers, female	Percent	9.3	NR	NR	
		Rate per 10,000 people, age standardized, male	Rate	73.1	NR	NR	
		Percentage of all cancers, male	Percent	7.5	NR	NR	
		Total	Count	4867.2	NR	NR	
Yabroff et al. [137]	Labor market participation	Job in past 12 months, compared to control group (45.9 % with a *p* value <0.001 for difference)	Percent	22.4	NR	15.6–29.3	6
	Sick leave	Days lost from wok due to health problems in past 12 months compared to control group (5.7 % with a *p* value <0.001 for difference)	Mean	10.0	NR	3.4–16.7	
	Presenteeism	Limited in work due to health issues compared to control group (17.6 % with a *p* value of <0.001 for difference)	Percent	32.4	NR	24.2–40.6	
Yaldo et al. [41]	Absenteeism	Mean higher absenteeism costs after 1 year of diagnosis compared to controls	USD	4245	NR	NR	7
*f*							
Bradley and Bednarek [85]	Unemployment	Unemployed 5–7 years after diagnosis cancer survivor	Percent	62.2	NR	NR	5
		Unemployed 5–7 years after diagnosis spouse of cancer survivor		51.3	NR	NR	
Costilla et al. [22]	DALYs	Female	Count	9334	NR	NR	NA
		% of all cancers (female)	Percent	14.3	NR	NR	
		Rate per 10,000 people (age standardised, female)	Rate	849	NR	NR	
		Male	Count	9806	NR	NR	
		% of all cancers (male)	Percent	15.9	NR	NR	
		Rate per 10,000 people (age standardised, male)	Rate	775	NR	NR	
Earle et al. [46]	Unemployment	Unemployment at 15 months	Percent	79	NR	NR	4
Fernandez de Larrea-Baz N et al. [95]	DALYs	Rate per 10,000 people (age standardised, female)	Rate	98	NR	NR	4
		Rate per 10,000 people (age standardised, male)	Rate	736	NR	NR	
		Rate per 10,000 people (age standardised, all)	Count	165,611	NR	NR	
Genova-Maleras et al. [4]	DALYs	Percentage of all causes of mortality	Percent	3.4	NR	NR	NA
		Rate per 1000 people, age standardized	Rate	3.8	NR	NR	
Molina et al. [111]	Return to work	Return to work at mean time since diagnosis(32.5 months)	Percent	15	NR	NR	5
Ohguri et al. [117]	Sick leave	Attendance rate after return to work of employees with disease compared to controls (*p* value 0.59)	Percent	75	NR	NR	4
Park et al. [48]	Labour market participation	Time until job loss	Cox PH	1.31	NR	1.12–1.53	7
		Time until re-employment (patients after job loss)	Cox PH	0.79	NR	0.55–1.16	
Park et al. [118]	Labour market participation	Cox proportional analysis comparing time until job loss between patients and controls	HR	2.22	NR	1.93–2.65	5
		Cox proportional analysis comparing time until re-employment between patients and controls	HR	0.45	NR	0.32–0.64	
Roelen et al. [122]	Return to work	Time to return to full-time work (days)	Count	484.0	NR	307–447	6
		Time to return to part-time work (days)	Count	377.0	NR	351–617	
Syse et al. [51]	Employment	Employment probability in year 2001 of cancer survivors compared to general population–men	OR	0.37	NR	0.31–0.45	6
		Employment probability in year 2001 of cancer survivors compared to general population–women	OR	0.58	NR	0.48–0.71	
Sjovall et al. [36]	Sick leave	Days	Count	275	NR	NR	5
Taskila-Brandt et al. [24]	Labor market participation	Employment status of cancer survivors 2–3 years post-diagnosis compared to general population (19 vs. 43 %)	RR	0.45	NR	0.34–0.59	6
Tevaarwerk et al. [43]	Unemployment	Unemployment	Percent	33	NR		6
Torp et al. [25]	Unemployment	Employment in 5 years from diagnosis (male)	OR	0.39	NR	0.18–0.83	9
		Employment in 5 years from diagnosis (female)	OR	0.39	NR	0.19–0.81	
Traebert et al. [21]	DALYs	Rate per 10,000 people, age standardized, female	Rate	87.6	NR	NR	NA
		Percentage of all cancers, female	Percent	9.8	NR	NR	
		Rate per 10,000 people, age standardized, male	Rate	239.9	NR	NR	
		Percentage of all cancers, male	Percent	24.5	NR	NR	
		Total	Count	10,832.2	NR	NR	
*g*							
Alexopoulos and Burdorf [54]	Sick leave	Days of sick leave during 2 year follow up attributable to COPD	Mean	8.53	NR	NR	2
Anesetti-Rothermel and Sambamoorthi [10]	Sick Leave	Work days in last year lost due to illness	Mean	8.600	0.76 (SE)	NR	6
Dacosta DiBonaventura et al. [53]	Productivity loss	Percentage reporting absenteeism (difference between cases of COPD and controls)	Percent	4.190	NR	NR	7
		Absenteeism hours (over last 7 days) (difference between COPD cases and controls)	Mean	1.250	NR	NR	
		Percentage reporting presenteeism (difference between cases of COPD and controls)	Percent	16.550	NR	NR	
		Estimated number of hours of presenteeism in last 7 days (difference between COPD cases and controls)	Mean	4.780	NR	NR	
		Percentage of those reporting work impairment (difference between cases of COPDand controls)	Percent	17.280	NR	NR	
		Percentage reporting absenteeism (difference between cases of COPD and controls)	Percent	2.330	NR	NR	
		Absenteeism hours (over last 7 days) (difference between cases of COPD and controls)	Mean	0.330	NR	NR	
		Percentage reporting presenteeism (difference between cases of COPD and controls)	Percent	10.230	NR	NR	
		Estimated number of hours of presenteeism in last 7 days (difference between cases of COPD and controls)	Mean	2.070	NR	NR	
		Percentage of those reporting work impairment (difference between cases of COPD and controls)	Percent	11.530	NR	NR	
Darkow et al. [63]	Productivity loss	Indirect per person per year	USD	9815	NR	8384–11246	6
Genova-Maleras et al. [4]	DALYs	Rate per 1000 age standardised	Rate	2.6	NR	NR	2
		Percentage of all causes of mortality	Percent	2.3	NR	NR	
Halpern et al. [98]	Productivity loss	Costs due to work loss up from 45 years up to age of retirement per patient per day	USD	100.55	NR	NR	6
		Days lost per patient of working age per year	Mean	18.7	NR	NR	
		Days lost per caregiver of working age per year	Mean	1.7	NR	NR	
	Unemployment	Unemployment due to condition	Percent	34	NR	NR	
Holden et al. [52]	Productivity loss	Absenteeism (no. of full/part days missed from work in last 4 weeks)	IRR	1.57	NR	1.33–1.86	3
		Presenteeism (self-rated score of overall performance in last 4 weeks)	IRR	1.22	NR	1.04–1.43	
Jansson et al. [59]	Productivity loss	Indirect per person per year	USD	749	NR	NR	6
Kremer et al. [55]	Unemployment	Percentage of who stopped work (among people in work) because of the onset of COPD	Percent	39	NR	NR	5
Leigh et al. [105]	Productivity loss	Total indirect costs in 1996 in billions of dollars	USD	21,400	NR	NR	3
Lokke et al. [62]	Unemployment	% receiving income from employment	Percent	16.7	NR	NR	7
	Productivity loss	Indirect costs per patient before the diagnosis	USD	4266	NR	NR	
		indirect costs per patient after diagnosis	USD	2816	NR	NR	
Lokke et al. [61]	Productivity loss	Indirect costs per patient before the diagnosis	USD	5912	NR	NR	9
		indirect costs per patient after diagnosis	USD	3819	NR	NR	
	Unemployment	% of spouses receiving income from employment	Percent	36.9	NR	NR	
Nair et al. [113]	Productivity loss	Short term 1 year productivity costs/per person	USD	527	NR	NR	9
		Absenteeism 1 year productivity costs/per person	USD	55	NR	NR	
		Total costs	USD		NR	NR	
Nishimura and Zaher [58]	Productivity loss	Modelled total annual costs per year in country (millions)	USD	1471	NR	NR	2
		Modelled indirect per patient	USD	262	NR	NR	
		Days modelled per person	Count	8.1	NR	NR	
Nowak et al. [60]	Productivity loss	early retirement (per patient/year) (all COPD stages)	USD	566	NR	NR	3
		early retirement (per patient/year) (light COPD)	USD	489	NR	NR	
		early retirement (per patient/year) (medium COPD)	USD	567	NR	NR	
		early retirement (per patient/year) (severe COPD)	USD	1064	NR	NR	
		disability (per patient/year) (all COPD stages)	USD	398	NR	NR	
		disability (per patient/year) (light COPD)	USD	459	NR	NR	
		disability (per patient/year) (medium COPD)	USD	249	NR	NR	
		disability (per patient/year) (severe COPD)	USD	340	NR	NR	
Orbon et al. [56]	Unemployment	Unemployment	Percent	53.8	NR	NR	4
Sin et al. [125]	Employment	Adjusted probability of being in work force for those with self-reported COPD compared to those without self-reported COPD	Percent	−3.9	NR	−1.3 to −6.4	4
	Productivity loss	Total loss productivity cost in 1994 in billions	USD	9.9	NR	NR	
Short et al. [124]	Unemployment	Limited amount of paid work possible due to illness (female)	OR	2.63	NR	2.03–3.42	5
		Limited amount of paid work possible due to illness (male)	OR	4.89	NR	3.46–6.9	
Strassels et al. [128]	Productivity loss	Number of lost work days COPD related	Mean	1.0	NR	<0.1–2.0	5
		Number of restricted activity days COPD related	Mean	15.9	NR	10.3–21.5	
van Boven et al. [57]	Productivity loss	Costs total per patient a year (2009)	USD	938	NR	NR	6
		Costs in total (2009)	USD	88,340,000	NR	NR	
	Absenteeism	Days total per patient (2009)	Count	10.7	NR	NR	
		Days total (2009)	Count	482,966	NR	NR	
Wang et al. [134]	Absenteeism	Annual excess in days	Mean	19.4	8.9 (SE)	NR	4
	Presenteeism	Annual excess in Days	Mean	27.5	15.6 (SE)	NR	
	Absenteeism & Presenteeism combined	Annual excess in days	Mean	42.9	17.0 (SE)	NR	
Ward et al. [135]	Unemployment	Inability to work attributable to COPD	Percent	10.6	NR	NR	6
	Productivity loss	Number work loss days per year	Mean	1.4	NR	NR	
*h*							
Helantera et al. [65]	Unemployment	Unemployed in 2007 for patients with dialysis or after kidney transplant	Percent	35	NR	NR	6
Klarenbach et al. [64]	Unemployment	Non-participation in labour force	OR	7.94	NR	1.6–39.43	6
*i*							
Adepoju et al. [71]	Absenteeism	Absenteeism Days total	Count	11,664	NR	NR	9
		Absenteeism Costs total	USD	85,314	NR	NR	
		Proportion of total productivity losses attributable to absenteeism	Percent	4	NR	NR	
		Days of reduced time at work as a sum of Inpatient and ambulatory visits	Count	7864	NR	NR	
		Costs of reduced time at work as sum of Inpatient and ambulatory visits	USD	866,744	NR	NR	
		Proportion of total productivity losses attributable to reduced time at work	Percent	3	NR	NR	
	Presenteeism	Presenteeism days total	Count	7864	NR	NR	
		Presenteeism Costs total	USD	866,744	NR	NR	
		Proportion of total productivity losses attributable to presenteeism	Percent	44	NR	NR	
	Productivity loss	Costs of premature mortality costs as a product of YLL and income	USD	953,373	NR	NR	
		Proportion of total productivity losses attributable premature mortality	Percent	49	NR	NR	
		Total productivity related loss	Count	20,064	NR	NR	
		Total productivity related costs loss	USD	1,962,314	NR	NR	
Alavinia and Burdorf [69]	Unemployment	Non participation in the labor force	OR	1.380	NR	0.990–1.930	4
Anesetti-Rothermel and Sambamoorthi [10]	Sick leave	Work days in last year lost due to illness	Mean	7.250	1.18 (SE)	NR	6
Bastida and Pagan [81]	Productivity loss	Unemployment due to diabetes In females	Maximum likelihood	−0.073	0.198	NR	NA
		Unemployment due to diabetes In males	Maximum likelihood	−1.047	0.447	NR	
Boles et al. [84]	Productivity loss	Lost earnings per diabetic person/week	USD	67	NR	NR	4
	Absenteeism	Absenteeism	OR	2.285	NR	1.167–4.474	
		Absenteeism	Least squares regression coefficient	3.254	7.286	NR	
	Presenteeism	Presenteeism	OR	1.271	NR	0.724–2.230	
		Presenteeism	Least squares regression coefficient	4.308	4.369	NR	
Bradshaw et al. [66]	DALYs	Total	Count	162,877	NR	NR	3
		Male	Count	102,454	NR	NR	
		Female	Count	101,690	NR	NR	
Burton et al. [91]	Presenteeism	Time management (work the required no. of hours; start work on time)	OR	1.401	NR	1.14–1.73	5
		Physical work activities (e.g. repeat the same hand motions; use work equipment)	OR	1.415	NR	1.15–1.75	
		Mental/interpersonal activities (concentration; teamwork)	OR	1.233	NR	1.02–1.50	
		Overall output (complete required amount of work; worked to capability)	OR	1.158	NR	0.95–1.42	
Collins et al. [92]	Productivity loss	Impairment score (WIS)	Count	17.8	NR	15.9, 19.6	7
		Absent hours per patient/month	Count	1.3	NR	0.6, 1.9	
		Work Impairment	Linear regression coefficient	−2.4	NR	NR	
		Absence	Logistic regression coefficient	1.2 (not significant)	NR	NR	
Dall et al. [68]	Productivity loss	Absenteeism	USD	2470	NR	NR	1
		Presenteeism	USD	18,715	NR	NR	
		Inability to work due to diabetes	USD	7276	NR	NR	
De Backer et al. [93]	Sick leave	Univariate analysis of high 1 year incidence rate of sick leave in diabetes compared to controls (25.3 %) in men (*p* value <0.001)	Percent	36.9	NR	NR	8
		Univariate analysis of long absences (defined as more than 7 days) in diabetes compared to controls (19.3 %) in men, (*p* value 0.002)	Percent	25.3	NR	NR	
		Univariate analysis for repetitive absences in diabetes compared to controls (14.5 %) in men (*p* value <0.001)	Percent	21.2	NR	NR	
		Adjusted analysis of high 1 year incidence rate of sick leave in diabetes compared to controls in men	OR	1.51	NR	1.22–1.88	
		Adjusted analysis of long absences in diabetes compared to controls in men	OR	1.11	NR	0.87–1.41	
		Adjusted analysis for repetitive absences in diabetes compared to controls in men	OR	1.54	NR	1.20–1.98	
		Univariate analysis of high 1 year incidence rate of sick leave in diabetes compared to controls (25.1 %) in women (*p* value <0.04)	Percent	33.9	NR	NR	
		Univariate analysis of long absences (defined as more than 7 days) in diabetes compared to controls (25.2 %) in women, (*p* value 0.04)	Percent	33.9	NR	NR	
		Univariate analysis for repetitive absences in diabetes compared to controls (24.0 %) in women (*p* value 0.002)	Percent	36.7	NR	NR	
		Adjusted analysis of high 1 year incidence rate of sick leave in diabetes compared to controls in women	OR	1.38	NR	0.89–2.14	
		Adjusted analysis of long absences in diabetes compared to controls in women	OR	1.45	NR	0.94–2.23	
		Adjusted analysis for repetitive absences in diabetes compared to controls in men	OR	1.71	NR	1.12–2.62	
Etyang et al. [6]	DALYs	Rate per 100,000 PY of observation	Rate	364	NR	NR	5
Fu et al. [97]	Productivity loss	Work loss days due to diabetes/year	Count	6.7	NR	NR	8
		Bed days due to diabetes/year	Count	13	NR	NR	
Genova-Maleras et al. [4]	DALYs	Rate per 1000 age standardised	Rate	2.2	NR	NR	2
		Percentage of all causes of mortality	Percent	1.9	NR	NR	
Herquelot et al. [100]	Presenteeism	Work disability due to diabetes	Incidence rate per 1000 person-years	7.9	NR	NR	7
		Work disability due to diabetes	HR	1.7	NR	1.0–2.9	
Holden et al. [52]	Productivity loss	Absenteeism, number of full/part days missed from work in last 4 weeks	IRR	1.17	NR	1.09–1.26	3
		Presenteeism, self-rated score of overall performance over last 4 weeks	IRR	0.89	NR	0.83–0.96	
Lenneman et al. [107]	Productivity loss	Productivity impairment	Unstandardized linear regression coefficient	1.816	NR	0.717–2.820	4
Klarenbach et al. [64]	Unemployment	Non-participation in labour force	OR	2.17	NR	1.2–3.93	6
Kessler et al. [70]	Productivity loss	Impairment days	Count	3.6	0.8	NR	2
		Any work impairment	OR	1.1	NR	0.6–1.9	
		Impairment days	Unstandardized linear regression coefficient	−0.3	0.5	NR	
Lavigne et al. [67]	Productivity loss	Work while feeling unwell	Percent	0.54	NR	NR	4
		Variance explained work efficiency losses	Percent	13	NR	NR	
		Hours of work lost due to diabetes, per month per person	Tobit regression coefficients	−1	NR	−13.92 to −12.18	
		Hours of absence from work due to diabetes, per month per person	Tobit regression coefficients	1	NR	−1.09 to −3.45	
		Hours of total productivity time lost per month per person due to diabetes	Tobit regression coefficients	8	NR	1.42–15.03	
		Cost of productivity time lost due to diabetes	Tobit regression coefficients	94	NR	−456.8 to −645.2	
Mayfield et al. [109]	Productivity loss	Work disability due to diabetes	Probit model estimates	1.46	0.228	NR	8
		Work disability due to diabetes	Percent	25.6	NR	NR	
		Work loss days due to diabetes	Linear regression	0.67	0.318	NR	
		Work loss days due to diabetes per year	Count	5.65	NR	NR	
		Lost earnings per diabetic person/year	USD	3099	NR	NR	
Robinson et al. [121]	Unemployment	Rate of unemployed in those economically active for males (controls 7.8 %)	Percent	21.9	NR	NR	7
		Rate of unemployed in those economically active for females (controls 5.1 %)	Percent	11.5	NR	NR	
		Rate of unemployed in those economically active for females (controls 7.0 % with a *p* value of <0.001 for difference)	Percent	18			
Short et al. [11]	Unemployment	Limited amount of paid work possible due to illness Female	OR	1.54	NR	1.23–1.92	5
		Limited amount of paid work possible due to illness Male	OR	2.02	NR	1.57–2.6	
Wang et al. [134]	Absenteeism	Annual excess in days	Mean	6.4	6.0 (SE)	NR	4
	Presenteeism	Annual excess in days	Mean	7.3	10.3 (SE)	NR	
	Absenteeism and Presenteeism combined	Annual excess in days	Mean	16.0	11.0 (SE)	NR	
*j*							
Torp et al. [25]	Unemployment	Unemployment at follow up	Percent	25.6	NR	NR	9
Earle et al. [46]	Unemployment	Unemployment at 15 months	Percent	69	NR	NR	4

*Cox PH* Cox proportional hazard regression, *DALY’s* disability adjusted life years, *IRR* incidence risk ratio, *NCD* no-communicable diseases, *NA* not applicable, *NR* not reported, *OR* odds ratio, *RR* relative risk, *SD* standard deviation, *USD* United States of America dollars

### Impact of stroke on productivity

Stroke accounted for 3.5 % of all DALYs reported in Spain (Table 2b) with a rate of 3.8 per 1000 people [4]. Another study from Spain reports a total count of DALYs of 418,052 with a higher number of male than for female (220,005 vs. 198,046) [13]. A study from Kenya reports a rate of 166 DALYs per 100,000 person-years observed [6]. In Western Australia, the average annual stroke-attributable DALY count is an estimated 26,315 for men and 30,918 for women [14]. In Spain, costs after diagnosis increased over time for caregivers but declined for patients (14,732 USD in caregivers compared to 2696 USD among patients after 1 year and 15,621 USD to 1362 USD after 2 years) [15]. Modeled productivity losses in South Korea were higher for a severe stroke among men (537,724 USD) than women (171,157 USD) [16]. A prospective surveillance study from Tanzania report a mean costs of productivity loss to be 213 USD [17]. Inconclusive evidence of the impact of stroke on RTW was reported. Estimates ranged from 26.7 to 75 % in studies reporting RTW in stroke patients after 1 year of the event [18, 19]. In Nigeria, 55 % returned to work at a mean of 19.5 months after stroke. A report from the United Kingdom (UK) found that 47 % were unemployed 1 year after stroke [20]. Increased odds to report limited ability for paid work were found among men (3.86) and women (2.26) after stroke [11].

### Impact of cervical cancer on productivity

There are strong regional differences in the percentage of DALYs attributable to cervical cancer (Table 2c) among women, from 1.6 % (absolute DALYs, 1061 per year) in New Zealand to 13.4 % (2516 per year) in Brazil [21, 22]. Cervical cancer patients in Argentina reported negative outcomes after 1 year; 45 % of patients reported reduced labor market participation, 28 % experienced work interruption and 5 % changed work [23]. Compared to the general population, the relative risk (RR) for cervical cancer survivors in labor force participation was 0.77 (95 % CI 0.67–0.90), 2–3 years after diagnosis in Finland [24]. In Norway however, no differences were found 5 years from diagnosis with an OR of 0.92 (0.63–1.34) [25].

### Impact of breast cancer on productivity

Of all the DALYs attributable to cancers among women, 27.3 % (17,840 per year) in New Zealand (Table 2d) and 13.4 % (6280 per year) in Brazil are attributable to breast cancer [21, 22]. Total mortality-related lifetime productivity loss costs in the USA were estimated to be 5.5 billion USD [26]. This was differentially distributed between the two ethnic groups reported, with 71 % (or 3.9 billion USD) of the costs attributable to white women and 24 % (or 1.3 billion) attributable to black women. Differential RTW and sick absence rates are also observed comparing black and white women in the USA; the percentage of white women returning to work three months after diagnosis was 74.2 % compared to 59.6 % of black women; the proportion reporting sick leave was 25.8 % of white women compared to 40.4 % of black women [27]. 1 year after primary surgery in Germany, nearly three times as many cancer survivors had left their job as compared to women in the control group. [28] Various studies suggest higher unemployment among breast cancer survivors, reported by around half after 1 year, 72 % after 2 years [29], 43 % after 6 years and 18 % after 9 years [27, 28, 30–32]. In contrast, in a study assessing unemployment among the spouses of breast cancer patients, no differences were found [33]. Differences between countries in average time to RTW were also found, from 11.4 months in the Netherlands [34] and 7.4 months in Canada [35] to only 3 months in Sweden [36]. Percentage of RTW after 1 year ranged from 54.3 % in a cross-sectional study from France to 82 % in a prospective study from the USA [37, 38].

### Impact of cancer on productivity

In New Zealand, of all the DALYs attributable to cancers, 12.9 % (8431 per year) among women and 13.5 % (8316 per year) among men are attributable to colon cancer (Table 2e) [22]. In Brazil, these proportions are 9.3 % among women and 7.5 % among men [21]. In Spain, 2.1 % of DALY’s overall are attributable to colon cancer [4]. In Iran the total burden of colorectal cancer in 2008 was 52,534 DALYs and higher for men than for women [39]. In the USA, annual productivity losses were calculated to be 20.9 billion USD [40], while costs due to absenteeism after 1 year of diagnosis was 4245 USD per patient compared to the general population [41]. Although the DALY and dollar costs of colon cancer are undoubtedly large, the evidence for micro-level labor market indicators including risk and proportions of RTW, sickness absence and employment following diagnosis and treatment is however inconclusive [25, 42–49]. In New Zealand, of all cancer-attributable DALYs, 14.4 % (9334 per year) among women and 15.9 % (9806 per year) among men are attributable to lung cancer (Table 2f) [22]. In Brazil, lung cancer results in an estimated 10,832 DALYs per year, 9.8 % of all cancer-related DALYs among women and 24.5 % among men [21]. In Spain, 3.4 % of all DALYs are attributable to lung cancer [4]. Most of the first year of disease (275 days) is spent in sickness absence in Sweden [36] and between 33 and 79 % of lung cancer patients in the USA were unemployed 15 months after diagnosis [43, 46]. Average time to re-enter the labor market was 484 days for full-time work and 377 for part-time work in the Netherlands [50]. The odds of re-entry into the labor market were significantly lower for lung cancer than the general population [24, 25, 51].

### Impact of COPD on productivity

COPD patients have a higher chance of working fewer hours, of absenteeism and of poorer work performance (presenteeism) (Table 2g). [11, 52, 53]. A COPD patient loses around 8.5 workdays per year due to disease [10, 54]. Between 39 and 50 % of people stopped working due to the onset of COPD in the Netherlands [55, 56]. COPD-related productivity losses cost the US economy around 88 million USD or around 482,966 working days per year [57]. Modeled annual costs of COPD, estimated at 1.47 billion USD [58], are higher in Japan than the USA. The productivity loss costs PP/PY were somewhat comparable between Germany, Sweden and the Netherlands (566, 749 and938 USD respectively) [57, 59, 60], but differed four-fold to estimated costs in Denmark (2816–3819 USD) [61, 62] and more than tenfold to what was estimated (9815 USD) in the USA [63]. In the USA, 8.5 work days are lost PP/PY on average [10], while COPD patients take an estimated 8.6 days of sickness absence in the Netherlands during a 2 year follow-up period [54]. Also in the Netherlands, 39 % of COPD patients left the labor force due to disease onset [55].

### Impact of chronic kidney disease on productivity

Only two studies (Table 2 h) examined the impact of CKD on productivity. One found that renal dysfunction was independently associated with labor force non-participation, with an odds ratio of 7.94 (95 % confidence interval, 1.60–39.43) [64]. The second study, evaluating labor market participation in CKD patients specifically after dialysis or transplantation, found that 35 % of these CKD patients were unemployed [65].

### Impact of diabetes mellitus on productivity

In Spain, nearly 2 % of all mortality-related DALYs are attributable to DM [4]. In South Africa, 162,877 DALYs annually are attributable to DM (Table 2i) [4, 66]. A study from Kenya reports a rate of 364 DALYs per 100,000 observed person-years [6]. An estimated 7.2 days are lost PP/PY due to DM in the USA [10] and DM patients have an increased risk of absenteeism, presenteeism and inability to work [4, 10, 11, 52, 64, 67–69]. Productivity days lost per year due to diabetes ranged from 3.6 to 7.3 [10, 70]. In the USA, proportion of productivity loss was large due to premature mortality (49 %) and presenteeism (44 %) compared to absenteeisim (4 %) and total productivity related costs were estimated to be 1,962,314 USD [71]. The odds of non-participation of the labor force for diabetes patients compared to the general population were slightly higher with borderline significance in the EU, an OR of 1.38 (95 % CI 0.99–1.93) [69].

## Discussion

This systematic review identified 126 studies investigating the impact of NCDs on productivity. Most studies (96 %) were from the Western world (North America, Europe or Asia Pacific), with limited evidence available from Brazil, South Africa, Kenya, Tanzania, Iran, Japan, South Korea and Argentina. Macro-economic productivity losses were measured in percentage and absolute numbers of DALYs and annual productivity loss costs (in USD). Studies also estimated productivity losses using labor market indicators including unemployment, RTW, absenteeism, presenteeism, sickness absence and loss in working hours. There is a clear scarcity in literature concerning the effect of CKD on productivity, with only two studies both reporting a substantial impact on productivity [64, 65].

### Diversity in the macroeconomic measures and outcomes

There were considerable global differences in the NCD-attributable DALY burden, especially the differential impact of each NCD comparing high-income countries (HIC) and low- and middle-income countries (LMIC). Lung and colon cancer account for nearly 30 % of all cancer-attributable DALYs in men in New Zealand whereas in Brazil, lung cancer alone accounts for nearly 25 %. Among women in HIC, breast cancer seems to impose a large productivity burden whereas cervical cancer impacts more dramatically in LMIC [4, 21, 22]. Although DALYs are a reliable measure and capture both years of life lost and years spent in ill-health, we found inconsistent application in the identified studies; some estimated proportions within specific disease groups or of the overall DALY burden in a country; others estimated absolute DALY numbers.

### Diversity in the macro-economic impact of the cardiopulmonary diseases

Absolute costs (measured in USD) were estimated for COPD, CHD, and stroke events [7, 9, 15, 57, 58, 71]. These studies mainly came from HIC, although two studies, one from Kenya and one from Tanzania, were also retrieved. In Australia, absenteeism and lower employment due to CHD cost 13.2 billion USD annually, as well as an additional 23 million USD in mortality-related costs [9]. Evidence suggests that COPD costs around 88 million USD or nearly 500,000 working days per year in the US compared to 1.47 billion (modeled) in Japan. While annual COPD-related productivity costs were comparable in Germany, Sweden and the Netherlands (between 566 and 938 USD), costs differed fourfold (2816–3819 USD) in Denmark, tenfold (9815 USD) in the USA [57, 59–63]. In the USA, nearly half of the annual 1.96 m USD productivity losses due to DM are attributable to mortality, with 44 % attributable to presenteeism and just 4 % to absenteeism In South Korea, modeled productivity losses for a stroke were 68 % higher among men compared to women [16]. Around half of all stroke survivors in unemployed after 1 year [20]. In Tanzania, productivity losses after 6 months following stroke were 213 USD on average although these losses were most acutely experienced by those in higher skill roles [17]. Interestingly, indirect productivity losses were higher among caregivers than stroke patients themselves and costs increased for caregivers but declined for patients after 1 and 2 years following a stroke in Spain. COPD patients experience reduced working hours, unemployment, absenteeism and presenteeism [10, 11, 52–56]. DM patients also have an increased risk of reduced labor market participation [10, 11, 52, 64]. By contrast, other than for absenteeism [10] the evidence for the risk of reduced labor market participation due to CVD is inconclusive. In Kenya, 68/100,000 person year observed are attributable to CVD compared to 166/100,000 for stroke and 364/100,000 for DM [6]. Although evidence is limited, the higher productivity impact associated with diseases with a large morbidity was perhaps to be expected; chronic diseases such as COPD and DM affect people during their productive years and cannot really be ‘cured’, only managed. The extent to which employers or societies support and enable NCD populations to remain members of the productive workforce will also differentially distribute the impact. The extent to which secondary or tertiary prevention is possible will also affect productivity estimates, specifically so for labor market indicators such as RTW, change in work status or unemployment.

### Diversity in the macroeconomic impact of cancer

Lung cancer survival is associated with reduced labor market participation through sickness absence, extended RTW [36, 50] and unemployment [25, 43, 46]. Total mortality-related lifetime productivity loss due to breast cancer were an estimated 5.5 billion USD in the USA [26] and annual productivity losses due to colon cancer costs the US economy 20.9 billion USD [40].We found inconclusive evidence of risk of reduced labor market participation (RTW, sickness absence and unemployment) following colon cancer diagnosis and treatment [25, 42–46, 48]. The evidence for breast cancer-related labor market drop-out shows higher unemployment among survivors 1, 2, 6 and 9 years after diagnosis [29–32]. Evidence from the USA also suggests ethnicity-patterned differences in sick leave and unemployment [27]. Along with possible socio-economic differences associated with these outcomes [72], pathophysiological differences may also play a role. African-American women have lower incidence of breast cancer but higher mortality and are also diagnosed in later stages and with more aggressive types of tumors [73]. However, we are cautious in over interpretation of this finding as few studies included ethnicity. Geographic differences in average months to RTW were observed from 11.4 in the Netherlands [34] to 7.4 in Canada [35] to just three months in Sweden [36].

Although evidence is limited, the higher productivity impact associated with diseases with a large morbidity was perhaps to be expected; chronic diseases such as COPD and DM affect people during their productive years and cannot really be ‘cured’, only managed. It is surprising that half of all productivity losses in the USA attributable to DM are due to mortality rather than absenteeism and presenteeism. The extent to which employers or societies support and enable NCD populations to remain members of the productive workforce will also differentially distribute the impact both within societies but also comparing more affluent to less affluent countries. The extent to which secondary or tertiary prevention is possible will also affect productivity estimates, specifically so for labor market indicators such as RTW, change in work status or unemployment.

### Comparison with the previous work

Findings of this systematic review generally concur with and further extend the previous reviews. This study is a comprehensive systematic review tackling work-related burden of six major NCDs using a global perspective and without language limitation. Two reviewers included and assessed the studies and references of the included studies were tracked for any missing evidence. These approaches ensured that we included most of the relevant articles in our review. Similar to previous reviews, we found that, due to a great amount of variation in the studies included, comparability and pooling the studies were not possible. Most of the previous reviews were performed non-systematically and previous systematic reviews have included studies only in English. Previous studies were mainly focused on the impact of cancers [74–78] on work-related outcomes (mainly RTW) and often included a mix of cancers without specifying the type of cancer. Van Muijen and colleagues [78] reviewed only cohort studies of cancer-related work outcomes and were focused on English language. Steiner and colleagues [76] reviewed English publications published up until 2003, Breton and colleagues were focused only on diabetes and Krisch and colleagues focused on COPD in Germany [79].

### Strengths and limitations of the current work

In this systematic review we evaluated the literature concerning the impact on productivity of six top NCDs. These six were selected based on their dominance in the global burden of disease and together make a huge contribution to mortality and morbidity worldwide. Several important issues are out of scope for this work but do merit future research. First, we did not look into the underlying mechanisms of what forces people with NCDs in and out of the labor force, specifically in terms of co-morbidities (certain NCDs cluster in the same populations) and financial/social means available at an individual and collective level. How these mechanisms interact will also be different according to the level of economic and social development. For example, children in LMIC are more likely to be forced into the labor market due to the onset of NCDs in parents compared to children in HIC and the productive output of this child cannot replace the loss due to drop out by the parents. These related topics should be addressed separately to better understand how to modify and target these outcomes more specifically. Second, we observed wide heterogeneity in all domains within the studies selected, including study design, methods and sources used to measure productivity, adjustment for confounders and analyses. Third, no identified studies quantified the differential productivity impact by national economic development and labor market structure across countries. How these inter-country macro-economic differences might mitigate or magnify productivity losses associated with NCDs is worth further exploration. Fourth, we identified a crucial gap of relevant information from LMICs—limiting the relevance of our review most acutely in these settings. This lack of evidence could reflect differences in disease burden, in research capacity, in welfare systems and in epidemiological surveillance. The burden of NCDs is growing rapidly in LMIC; countries that often lack capacity in these key areas of support, prevention and knowledge generation. Further evaluation, therefore, of the macro-economic impact in the LMIC countries is urgently needed. Also, many NCDs affect people cumulatively over time; people may suffer DM, may experience absenteeism/presenteeism as a result, may reduce work as DM worsens and may finally drop out of the workforce due a stroke or CHD, which is related to the DM. Given NCDs are shifting more and more into chronic conditions, as our understanding of treatment and natural history improve, it would be of great interest to investigate the effects over the life course rather than using short time horizons such as a year. This is no mean feat, but could be crucial for developing a better understanding of the economic impact of NCDs on a regional, national and international level. Also out of scope for this review but of interest for future work are the productivity-related impact of behavioural risk factors that contribute to the development of NCDs.

## Conclusions

In summary, available studies indicate that the six main NCDs generate a large impact on macro-economic productivity in the WHO regions. However, this evidence is heterogeneous, of varying quality and not evenly geographically distributed. Data from LMI countries in economic and epidemiological transition are virtually absent. Further work to reliably quantify the absolute global impact of NCDs on macro-economic productivity and DALYs is urgently required.

### Electronic supplementary material

Supplementary material 1 (DOC 94 kb)

## Appendix 1: Search strategy up to 6th of November 2014

(‘non communicable disease’/de OR ‘ischemic heart disease’/exp OR ‘cerebrovascular accident’/exp OR ‘chronic obstructive lung disease’/de OR ‘lung cancer’/exp OR ‘colon cancer’/exp OR ‘breast cancer’/exp OR ‘chronic kidney disease’/de OR ‘non insulin dependent diabetes mellitus’/de OR ‘uterine cervix cancer’/exp OR (‘non communicable’ OR noncommunicable OR ((heart OR cardiac OR cardial OR cardiopath* OR cardiomyopath* OR coronar* OR myocard*) NEAR/3 (ischem* OR ischaem* OR anoxia OR hypoxia)) OR (coronary NEAR/3 (insufficien* OR occlus* OR disease* OR acute OR atherosclero* OR arteriosclero* OR sclero* OR cardiosclero* OR constrict* OR vasoconstrict* OR obstruct* OR stenosis* OR thrombo*)) OR angina* OR ((heart OR myocard* OR cardiac OR cadial) NEAR/3 infarct*) OR ((cerebrovascul* OR brain OR ‘cerebral vascular’ OR ‘cerebro vascular’) NEAR/3 (accident* OR lesion* OR attack OR ischem* OR ischaem* OR insult* OR insuffucien* OR arrest* OR apoplex*)) OR cva OR stroke OR (chronic AND (obstruct* NEAR/3 (lung* OR pulmonar* OR airway* OR bronch* OR respirat*))) OR ((lung* OR pulmonar* OR colon* OR colorect* OR breast* OR mamma*) NEAR/3 (neoplas* OR cancer* OR carcino* OR adenocarcino* OR metasta* OR sarcom*)) OR (chronic NEAR/3 (kidney* OR nephropathy* OR renal)) OR ((‘adult onset’ OR ‘type 2’ OR ‘type ii’ OR ‘non-insulin dependent’ OR ‘noninsulin dependent’ OR ‘insulin independent’) NEAR/3 diabet*) OR ((cervix OR cervical) NEAR/3 (cancer* OR neoplas* OR tumo* OR carcinom* OR malign*))):ab,ti) AND (adult/exp) AND (‘randomized controlled trial’/exp OR ‘cohort analysis’/de OR ‘case control study’/exp OR ‘cross-sectional study’/de OR ‘systematic review’/de OR ‘meta analysis’/de OR ecology/exp OR ‘ecosystem health’/exp OR ‘ecosystem monitoring’/exp OR model/exp OR ((random* NEAR/3 (trial* OR control*)) OR rct* OR cohort* OR ‘case control’ OR ‘cross-sectional’ OR (systematic* NEAR/3 review*) OR metaanaly* OR (meta NEXT/1 analy*) OR ecolog* OR ecosystem* OR model*):ab,ti) NOT ([animals]/lim NOT [humans]/lim) NOT ([Conference Abstract]/lim OR [Conference Paper]/lim OR [Letter]/lim OR [Note]/lim OR [Conference Review]/lim OR [Editorial]/lim OR [Erratum]/lim).

AND (productivity/de OR absenteeism/de OR ‘job performance’/de OR ‘return to work’/de OR ‘work capacity’/de OR ‘working time’/de OR ‘medical leave’/de OR workload/de OR retirement/de OR employment/exp OR unemployment/de OR (productivit* OR unproductivit* OR absenteeis* OR presenteeis* OR ((job OR work* OR profession* OR occupation* OR labour) NEAR/3 (perform* OR efficien* OR return* OR back OR capacit* OR abilit* OR disabilit* OR unab* OR limit* OR impair* OR loss OR losing OR restrict* OR reduct* OR input*)) OR (work* NEXT/1 (time OR week* OR day* OR load*)) OR workweek* OR workday* OR ((medical OR sick) NEXT/1 leave) OR workload* OR ‘time off work’ OR retire* OR employment* OR employed* OR unemploy* OR daly OR (‘disability adjusted’ NEXT/2 year*)):ab,ti).

## Appendix 2: Newcastle–ottawa quality assessment scale

### Cross-sectional and descriptive studies

*Note*: A study can be awarded a maximum of one star for each numbered item within the Selection and Exposure categories. A maximum of two stars can be given for Comparability.

Selection

1. Is definition of NCDs adequate?
  - Yes, according to a clear and widely used definition*****
  - Yes, e.g. record linkage or based on self-reports
  - No description
2. Representativeness of the cases
  - Consecutive or obviously representative series of cases*****
  - Excluded cases are random*****
  - No description of the excluded cases or potential for selection biases or not stated
3. Comparison with a reference group
  - The results are compared with a reference from community or with the status of the cases prior to the disease*****
  - The results are compared with the results from other patients
  - No description/no comparison available
4. Definition of reference
  - Individuals with no NCD or sample from general population or the same individuals before NCD suffering*****
  - Non community comparator is described
  - No description of source

### Comparability

1. Comparability of the results on the basis of the design or analysis
  - The results are described in age and sex sub groups (sex is not applicable for female diseases)*****
  - The results are additionally *adjusted for/described in* different socioeconomic factors or disease related confounders*****

### Exposure (costs, productivity, households)

1. Ascertainment of exposure
  - Secure record (e.g. surgical records, hospital records, and administrative records, national…)*****
  - Structured interview where blind to case/control status*****
  - Interview not blinded to case/control status
  - Written self-report or medical record only
  - No description
2. Same method of ascertainment for NCDs and comparators
  - Yes*****
  - No
  - No comparator group exist
3. Non-response rate
  - All participants included or same rate for both groups or respondents and non-respondents have the same characteristics*****
  - Non respondents described
  - Rate different and no designation
  - Response rate not described
