# Supplementary material for: The global impact of non-communicable diseases on macro-economic productivity: a systematic review
Source: Eur J Epidemiol. 2015 Apr 3;30(5):357–95. doi: 10.1007/s10654-015-0026-5 (PMC4457808; doi:10.1007/s10654-015-0026-5)
Supplement: Supplementary file 1 — Supplementary material 1 (DOC 94 kb) [file 10654_2015_26_MOESM1_ESM.doc]

**Appendix 3.** PRISMA checklist

| **Section/topic** | **#** | **Checklist item** | **Reported on page #** |
| --- | --- | --- | --- |
| **TITLE** | | |  |
| Title | 1 | Identify the report as a systematic review, meta-analysis, or both. | 1,3 |
| **ABSTRACT** | | |  |
| Structured summary | 2 | Provide a structured summary including, as applicable: background; objectives; data sources; study eligibility criteria, participants, and interventions; study appraisal and synthesis methods; results; limitations; conclusions and implications of key findings; systematic review registration number. | 3 |
| **INTRODUCTION** | | |  |
| Rationale | 3 | Describe the rationale for the review in the context of what is already known. | 5 |
| Objectives | 4 | Provide an explicit statement of questions being addressed with reference to participants, interventions, comparisons, outcomes, and study design (PICOS). | 5 |
| **METHODS** | | |  |
| Protocol and registration | 5 | Indicate if a review protocol exists, if and where it can be accessed (e.g., Web address), and, if available, provide registration information including registration number. | NA |
| Eligibility criteria | 6 | Specify study characteristics (e.g., PICOS, length of follow-up) and report characteristics (e.g., years considered, language, publication status) used as criteria for eligibility, giving rationale. | 6 |
| Information sources | 7 | Describe all information sources (e.g., databases with dates of coverage, contact with study authors to identify additional studies) in the search and date last searched. | 6 |
| Search | 8 | Present full electronic search strategy for at least one database, including any limits used, such that it could be repeated. | 6 and Appendix 1 |
| Study selection | 9 | State the process for selecting studies (i.e., screening, eligibility, included in systematic review, and, if applicable, included in the meta-analysis). | 6, 7 |
| Data collection process | 10 | Describe method of data extraction from reports (e.g., piloted forms, independently, in duplicate) and any processes for obtaining and confirming data from investigators. | 7 |
| Data items | 11 | List and define all variables for which data were sought (e.g., PICOS, funding sources) and any assumptions and simplifications made. | 7 |
| Risk of bias in individual studies | 12 | Describe methods used for assessing risk of bias of individual studies (including specification of whether this was done at the study or outcome level), and how this information is to be used in any data synthesis. | 7 |

| **Section/topic** | **#** | **Checklist item** | **Reported on page #** | |
| --- | --- | --- | --- | --- |
| Summary measures | 13 | State the principal summary measures (e.g., risk ratio, difference in means). | 7,8 | |
| Synthesis of results | 14 | Describe the methods of handling data and combining results of studies, if done, including measures of consistency (e.g., I^2^) for each meta-analysis. | 7,8 | |
| Risk of bias across studies | 15 | Specify any assessment of risk of bias that may affect the cumulative evidence (e.g., publication bias, selective reporting within studies). | 7,8 | |
| Additional analyses | 16 | Describe methods of additional analyses (e.g., sensitivity or subgroup analyses, meta-regression), if done, indicating which were pre-specified. | NA | |
| **RESULTS** | | | |  |
| Study selection | 17 | Give numbers of studies screened, assessed for eligibility, and included in the review, with reasons for exclusions at each stage, ideally with a flow diagram. | | 9 and Figure 1 |
| Study characteristics | 18 | For each study, present characteristics for which data were extracted (e.g., study size, PICOS, follow-up period) and provide the citations. | | 9 and Table 1 |
| Risk of bias within studies | 19 | Present data on risk of bias of each study and, if available, any outcome level assessment (see item 12). | | 9 and Table 2 |
| Results of individual studies | 20 | For all outcomes considered (benefits or harms), present, for each study: (a) simple summary data for each intervention group (b) effect estimates and confidence intervals, ideally with a forest plot. | | 9-15, Tables 1 and 2 |
| Synthesis of results | 21 | Present results of each meta-analysis done, including confidence intervals and measures of consistency. | | NA |
| Risk of bias across studies | 22 | Present results of any assessment of risk of bias across studies (see Item 15). | | Table 2 |
| Additional analysis | 23 | Give results of additional analyses, if done (e.g., sensitivity or subgroup analyses, meta-regression [see Item 16]). | | NA |
| **DISCUSSION** | | | |  |
| Summary of evidence | 24 | Summarize the main findings including the strength of evidence for each main outcome; consider their relevance to key groups (e.g., healthcare providers, users, and policy makers). | | 16-20 |
| Limitations | 25 | Discuss limitations at study and outcome level (e.g., risk of bias), and at review-level (e.g., incomplete retrieval of identified research, reporting bias). | | 19 |
| Conclusions | 26 | Provide a general interpretation of the results in the context of other evidence, and implications for future research. | | 20 |
| **FUNDING** | | | |  |
| Funding | 27 | Describe sources of funding for the systematic review and other support (e.g., supply of data); role of funders for the systematic review. | | 21 |

**Appendix 4:** MOOSE checklist

| **Criteria** | | **Brief description of how the criteria were handled in the meta-analysis** |
| --- | --- | --- |
| **Reporting of background should include** | |  |
| √ | Problem definition | Non-communicable diseases are currently the leading causes of adult death and disability worldwide and the global burden is expected to rise. However, little work has been done to systematically review the current literature on the economic burden of NCDs globally. |
| √ | Hypothesis statement | NCDs pose a significant burden on macro-economic productivity, which is likely to increase over time. |
| √ | Description of study outcomes | We included studies that estimated the impact of at least one of the six major NCDs, on at least one measure of macro-economic productivity, including: DALYs, economic costs related to reduced work productivity, absenteeism, presenteeism, (un)employment, (non-) return to work (RTW) after sickness absence and medical/sick leave. |
| √ | Type of exposure or intervention used | Six major non-communicable diseases including coronary heart disease, stroke, type 2 diabetes mellitus, cancer (lung, colon, cervical and breast), chronic obstructive pulmonary disease and chronic kidney disease. |
| √ | Type of study designs used | Eligible study designs included randomized controlled trials (RCTs), cohort, case-control, cross-sectional, systematic reviews, ecological studies and modelling studies. |
| √ | Study population | Only studies carried out in adults (>18 years old) were included. |
| **Reporting of search strategy should include** | |  |
| √ | Qualifications of searchers | The credentials of the investigators are indicated in the authors list. |
| √ | Search strategy, including time period included in the synthesis and keywords | Search strategy and time periods are detailed in page 7 of the manuscript and in Figure 1 and the full search strategy is available in appendix 1. |
| √ | Databases and registries searched | Medline, Embase and Google Scholar |
| √ | Search software used, name and version, including special features | We did not employ a search software. Endnote was used to merge retrieved citations and eliminate duplications |
| √ | Use of hand searching | We hand-searched bibliographies of retrieved systematic reviews and meta-analysis for additional references. |
| √ | List of citations located and those excluded, including justifications | Details of the literature search process are outlined in the flow chart. Citations for the included studies are included in the text and table 1. The citation list for excluded studies is available upon request. |
| √ | Method of addressing articles published in languages other than English | We placed no restrictions on language; local translation services were available |
| √ | Method of handling abstracts and unpublished studies | Systematic reviews were used to identify further references. Authors of included studies were contacted to retrieve missing full texts and to identify any missing studies. |
| √ | Description of any contact with authors | Authors of included studies were contacted to retrieve missing full texts and to identify any missing studies. |
| **Reporting of methods should include** | |  |
| √ | Description of relevance or appropriateness of studies assembled for assessing the hypothesis to be tested | Detailed inclusion and exclusion criteria were described in the methods section. |
| √ | Rationale for the selection and coding of data | A predesigned data collection form was prepared to extract the relevant information from the included full texts, including study design, WHO region, characteristics of the study participants, NCDs details and economic measures reported |
| √ | Assessment of confounding | We performed qualitative analyses to evaluate differences between studies |
| √ | Assessment of study quality, including blinding of quality assessors; stratification or regression on possible predictors of study results | We used the Newcastle- Ottawa Scale (NOS) to evaluate the quality of cross-sectional, case-control and cohort studies included in this review |
| √ | Assessment of heterogeneity | We were not able to pool due to large levels of heterogeneity evaluated visually and statistically |
| √ | Description of statistical methods in sufficient detail to be replicated | We conducted qualitative analysis of the data. Due to large heterogeneity between studies we solely performed qualitative analyses of the data. |
| √ | Provision of appropriate tables and graphics | We included 1 main figure, 2 main tables, and 4 appendices |
| **Reporting of results should include** | |  |
| √ | Graph summarizing individual study estimates and overall estimate | The heterogeneity of the outcomes across the different studies thwarted our ability to provide pooled estimations. |
| √ | Table giving descriptive information for each study included | Tables 1 and 2 |
| √ | Results of sensitivity testing | The heterogeneity of the outcomes across the different studies thwarted our ability to provide pooled estimations. |
| √ | Indication of statistical uncertainty of findings | 95% confidence intervals or SD’s were presented if available |
| **Reporting of discussion should include** | |  |
| √ | Quantitative assessment of bias | Not applicable |
| √ | Justification for exclusion | We excluded studies that had no or unclear definition of exposure and outcome, or data extraction was not feasible. |
| √ | Assessment of quality of included studies | We used the Newcastle- Ottawa Scale (NOS) to evaluate the quality of cross-sectional, case-control and cohort studies included in this review |
| **Reporting of conclusions should include** | |  |
| √ | Consideration of alternative explanations for observed results | Due to the lack of standardisation of measurements of macro-economic productivity, it is difficult to provide unifying statements within a larger number of manuscripts hampered by large levels of heterogeneity. |
| √ | Generalization of the conclusions | The generalizability of our findings has been enhanced by the involvement of data from 4 WHO regions, including region of the Americas, Europe, West Pacific Region and the African Region. However there is a clear lack of evidence from South East Asia and the Middle East. |
| √ | Guidelines for future research | Further work is necessary to standardize the methods to consistently assess the economic impact of NCDs worldwide and to involve hitherto under-addressed low and middle income populations across the globe. |
| √ | Disclosure of funding source | Completion of this manuscript was supported by a grant from the WHO. |
